# Psychopathology, Personality and Depression after Acute Coronary Syndrome: A Network Analysis in an Italian Population

**DOI:** 10.3390/diagnostics13050915

**Published:** 2023-02-28

**Authors:** Federica Folesani, Lorenzo Luviè, Cristina Palazzi, Carlo Marchesi, Rodolfo Rossi, Martino Belvederi Murri, Paolo Ossola

**Affiliations:** 1 Department of Neuroscience and Rehabilitation, Institute of Psychiatry, University of Ferrara, 44121 Ferrara, Italy; 2 Department of Medicine and Surgery, University of Parma, 43121 Parma, Italy; 3 Department of Mental Health, AUSL of Parma, 43125 Parma, Italy; 4 Department of Biotechnological and Applied Clinical Sciences, Section of Psychiatry, University of L’Aquila, 67100 L’Aquila, Italy; 5 Department of System Medicine, Section of Psychiatry, University of Rome Tor Vergata, 00133 Rome, Italy

**Keywords:** acute coronary syndrome, personality, network analysis, depression, risk factors

## Abstract

Several biopsychosocial factors are associated with the onset of a Major Depressive Episode (MDE) after cardiovascular events. However, little is known of the interaction between trait- and state-like symptoms and characteristics and their role in predisposing cardiac patients to MDEs. Three hundred and four subjects were selected among patients admitted for the first time at a Coronary Intensive Care Unit. Assessment comprised personality features, psychiatric symptoms and general psychological distress; the occurrences of MDEs and Major Adverse Cardiovascular Events (MACE) were recorded during a two-year follow-up period. Network analyses of state-like symptoms and trait-like features were compared between patients with and without MDEs and MACE during follow-up. Individuals with and without MDEs differed in sociodemographic characteristics and baseline depressive symptoms. Network comparison revealed significant differences in personality features, not state-like symptoms: the group with MDEs displayed greater Type D personality traits and alexithymia as well as stronger associations between alexithymia and negative affectivity (edge differences between negative affectivity and difficulty identifying feelings was 0.303, and difficulty describing feelings was 0.439). The vulnerability to depression in cardiac patients is associated with personality features but not with state-like symptoms. Personality evaluation at the first cardiac event may help identify individuals more vulnerable to development of an MDE, and they could be referred to specialist care in order to reduce their risk.

## 1. Introduction

Vulnerability to depression encompasses an intricate web of interactions between sociodemographic factors, personality, psychological states and negative life events. The case of depression following acute cardiac events illustrates the level of complexity of such interactions and the involvement of psychological, cognitive and biological mechanisms. Network analyses may be helpful to highlight the implicated factors and the most powerful links between them, leading to new prospects for both clinical and research trajectories.

Acute Coronary Syndrome (ACS) is a prototypical example of a stressful event that may trigger a depressive episode in vulnerable individuals. As many as 20% of patients with ACS develop major depressive disorder, and almost two-thirds of ACS patients still present significant depressive symptoms months after the event [1]. In addition, the onset of depressive symptoms is associated with an increased risk of developing Coronary Heart Disease (CHD) [2] and a worse clinical course of pre-existing CHD [3,4]. Ultimately, depression is associated with an increase of the recurrence of cardiac events and the risk of cardiac-related mortality [4,5,6,7]. The putative causal mechanisms of interaction between depression and CHD are complex and bi-directional, involving alterations of cardiovascular biohumoral [7,8,9] and neurobiological homeostasis [7,10], non-adherence to medications and to cardiac rehabilitation [11,12,13,14], detrimental lifestyles (e.g., smoking, sedentary behavior, etc.) [15,16,17] and the use of medications such as antipsychotics [18].

Sociodemographic features, stable (trait-like, pre-existing) personality features and incidental (state-like, time-related) emotional symptoms could be associated with depression in ACS, such as younger age [19], female gender [20,21], widowhood [6], low education [22] and social isolation [19,23]. In addition, depression is more frequent among those with Type D personalities [24,25,26], higher levels of neuroticism [6,27,28], novelty-seeking behavior, harm avoidance [6], narcissistic traits [6], lower self-directedness [29] and, possibly, alexithymia [30,31,32]. Recently, studies highlighted a link between the onset of specific symptoms after ACS and the subsequent development of depression, such as exhaustion, fatigue, poor sleep quality [21,28] and anhedonia [6]. trait- and state-like psychological constructs are, in fact, heavily inter-dependent [6,33,34,35,36], as it is in the case of alexithymia [36,37,38,39] or Type D personalities [40,41,42].

Few studies, to our knowledge, have so far examined the interactions of multiple psychosocial risk factors for depression in ACS. Focusing on specific risk factors could be helpful in understanding the etiological mechanisms that underlie the association between depression and coronary disease, clarifying the state- and trait-like factors of vulnerability. It could be useful in identifying possible specific pathways in the progression of symptoms in order to promote early diagnosis and well-timed intervention strategies aimed at avoiding the development of full-blown psychopathology through more specific therapeutic targets. Network analyses may represent a strategic tool to explore multiple reciprocal causal relationships between symptoms and personality features at the same time [43,44]. The network approach to psychopathology, in fact, assumes that symptoms and signs of mental disorders may cause each other through multiple complex interactions, rather than having a single explanatory underlying mechanism [45]. External events, such as stressful life events or physical illnesses, may trigger a depressive episode by “activating” one or more symptoms, which, in turn, may cause other manifestations of the disorder with feed-forward or feedback loops. A state of chronic activation may depend ultimately on the interaction between external factors and individual pathways of vulnerability (e.g., a propensity of specific symptoms in the network to remain active). Network analyses are used to detect the conditional dependencies between variables (“nodes”) and represent them as “edges” of varying strength. 

Thus, the aim of this study was to investigate vulnerability towards depression in a sample of subjects at their first ACS event by examining the interaction between personality traits and psychological symptoms. Specifically, we aimed at identifying baseline clinical and sociodemographic features that could predict the onset of depression in the first two years since the first acute cardiac event. The secondary aim of the study was to evaluate features that were associated with the recurrence of coronary disease or death due to cardiovascular events. 

## 2. Methods

### 2.1. Participants

We recruited consecutive patients admitted to the Coronary Intensive Care Unit of the University Hospital of Parma for Acute Coronary Syndrome (ACS) between January 2009 and September 2012 that met the following inclusion criteria. Patients enrolled were presenting ACS symptoms for the first time; specifically, these symptoms were: a ST-segment elevation myocardial infarction (STEMI), a non-ST-segment elevation myocardial infarction (NSTEMI) or unstable angina [46,47]. The working diagnosis of NSTEMI ACS was a rule-out diagnosis based on ECG readings. Biomarkers (troponins) further distinguished NSTEMIs and unstable anginas [46].

Eligibility criteria were as follows: (a) first episode of Acute Coronary Syndrome (ACS)—patients were excluded if they presented previous cardiovascular diseases; (b) being over 18 years old; (c) being native or proficient in Italian; (d) having no signs of cognitive impairment as shown by achieving a Mini Mental State Examination score greater than 25 [48]; (e) having no history of substance abuse or dependence according to the DSM-IV-TR [49]; (f) having no history of Major Depressive Disorder (MDD) or any current Major Depressive Episode (MDE) at baseline according to the DSM-IV-TR [49]; (g) not taking any psychotropic medication or having any diagnosis of a schizophrenia spectrum disorder according to the DSM-IV-TR [49]. 

The study was approved by the local ethics committee and conducted according to the Declaration of Helsinki. Informed consent was provided by all participants after the study was completely explained. 

### 2.2. Baseline Assessment 

This study had a case-control longitudinal design comprising a baseline assessment and seven other assessments (at months one, two, four, six, nine, twelve and twenty-four).

At baseline, participant sociodemographic and clinical characteristics were recorded, including height, weight and alcohol and smoking habits. 

The Global Registry of Acute Coronary Events (GRACE) score [50] was computed to assess mortality risk after acute coronary events within six months of hospital discharge. The GRACE score takes into account age, previous history of MI, heart rate, systolic blood pressure, serum creatinine, cardiac enzymes, the presence of congestive heart failure, ST-segment depression and in-hospital percutaneous intervention. The score ranges from 1 to 263 points, and higher scores correspond to a greater risk of mortality. 

The assessment of personality and psychopathology included both interviews and self-reported questionnaires. To evaluate state-like symptoms, participants were assessed with the Primary Care Evaluation of Mental Disorder (PRIME-MD, an interview to establish the presence of MDD), the PRIME-MD Patient Health Questionnaire (PRIME-MD PHQ, the first self-report version of PRIME-MD, which is able to rate the presence and severity of different disorders from the DSM-IV) and the Hospital Anxiety and Depression Scale (HADS, a 14-item self-report questionnaire that rates the severity of anxiety and depression). To evaluate stable trait-like characteristics, participants were assessed with the Temperament and Character Inventory (TCI, a self-report scale which describes temperament and character), the Type D Personality Scale (DS-14, a self-report questionnaire used to assess specific dimensions such as negative affectivity and social inhibition), the Toronto Alexithymia Scale, 20-item version (TAS-20, self-report instrument to rate the presence of alexithymia) and the Defense Style Questionnaire (DSQ-40, 40-item version, which describes the maturity and functionality of the patient’s defensive mechanisms).

Not all subscales or modules used in the assessment were considered for the analysis. For state-like symptoms, the PRIME-MD, PRIME-MD PHQ were used, except for questions about menstruation and psychosocial stressors and we used the subscales of anxiety (HADS-A) and depression (HADS-D) from HADS. The stable trait-like characteristic subscales from TCI used in this study were novelty-seeking (NS), harm avoidance (HA), reward dependence (RD), self-directedness (SD), cooperativeness (C) and self-transcendence (ST) (i.e., all except for the persistence subscale); from DS-14, the subscales used were negative affectivity (NA) and social inhibition (SI); from DSQ-40, the subscales used were mature, immature and neurotic; from TAS-20, the subscales used were identifying feelings (DIF), difficulty describing, communicating and expressing feelings to others (DDF) and externally oriented thinking (EOT).

### 2.3. Follow-Up Assessments and Outcomes

Patients were reassessed with the same questionnaires seven times, namely after months one, two, four, six, nine, twelve and twenty-four since enrollment in the study (except for the TCI, which was given at baseline only). A two-year follow-up period was used considering the high incidence of depression within this time frame after an ACS event [51]. At each time point, the occurrence of an MDE was ascertained by considering the PRIME-MD score, and it was further confirmed with an interview performed by an expert psychiatrist to evaluate if the answers were appropriate to the patients’ conditions. 

In addition, we recorded reoccurrences of Major Adverse Cardiovascular Events (MACE), defined as recurrences of ACS events, nonelective revascularization, acute hospitalization because of postischemic heart failure or death due to cardiac causes [52]. The definition of MACE did not include all-cause mortality and strokes, as the mechanisms of association with depression and their predictors might differ among these groups [53]. Information on MACE were registered by the Coronary Intensive Care Unit Technology registry. If they occurred and were thereby treated outside of the study catchment, then they were intercepted by phone calls to the participants. Further details on the MACE follow-up methodology are available in [53,54]. 

## 3. Data Analysis

To identify the psychosocial risk factors for depression after ACS, we first compared the baseline characteristics of participants who developed a Major Depressive Episode during the follow-up period with those of subjects who did not develop depression. Similarly, we sought to identify the risk factors for ACS recurrence by comparing subjects who went on to have another MACE or die by cardiac-related causes with those who remained free from cardiac recurrences. Analyses focused on the comparison of baseline sociodemographic characteristics, state symptom severity and levels of personality traits endorsed by participants using univariate statistics, namely the Wilcoxon rank-sum test, Pearson’s chi-squared test, and Fisher’s exact test. Moreover, we compared the strength of the associations between different clinical features using network analyses. 

### 3.1. State Characteristics and Data Reduction

Multiple rating scale items were available to rate the same anxiety, depressive and somatic symptoms (i.e., HADS, PRIME-MD and PHQ). This is advantageous because it limits the impact of measurement error [55]; however, using redundant items in network analyses may bias the estimation of symptom associations. Thus, data analyses were preceded by data reduction, namely by combining similar symptoms into fewer dimensions. This was shown to improve the stability of network analyses and reduce the redundancy of nodes [56]. Exploratory Graph Analysis (EGA) is a community detection approach that identifies strongly inter-related variables and accounts for their reciprocal causal relationships [57]. This method yielded good fits to the data in previous studies on depression [58]. The walktrap algorithm was used to identify “symptom complexes” of inter-related symptoms by performing random walks in the network of items of the depression, anxiety and somatic symptoms of the baseline sample [59,60]. Subsequently, we computed the score of each symptom complex for each individual as the sum of ratings of symptoms belonging to the same complex, which was weighted by their dominant network loading. Network loadings measure the importance of items within a symptom complex [61]. Analyses were performed with the *EGAnet* package version 0.9.4 [62]. 

### 3.2. Network Estimation

We used network analyses to estimate the strength of associations between (a) psychopathological state-like symptoms and (b) personality trait-like features. The psychopathological symptom network comprised data of symptom complex scores derived from the PRIME-MD, HADS and PRIME-MD PHQ results. The network of personality estimated the relationships between the subscales of the TCI, TAS-20, DS-14 and DSQ-40. For each analysis, we first conducted explorative analyses in the whole sample (reported in the Supplementary Material). Then, we compared the networks of participants with and without depression or MACE at follow-up in terms of the number and composition of symptom complexes. Finally, we compared the global strengths of the networks and the strength of each association between symptoms/personality traits (edges). 

Network analyses were conducted using a Bayesian approach, as developed in the 2.0.3 BGGM R package [63,64]. Posterior Credibility Intervals (CrI) were used to estimate each pairwise association between symptoms (edge strength). We tested if these parameters were non-zero using 90% CrI. In addition, we estimated the differences between global network strengths and between each edge strength across different levels of outcomes (depressed vs. non-depressed; MACE vs. no MACE) using a 90% confidence level. Networks were displayed using the qgraph 1.6.5 version R package [65]. The values of the edge weights are reported in the Weighted Adjacency Matrices (WAMs). Each node in the network corresponds to a variable. Green edges connecting the nodes indicate positive correlations, and red edges indicate negative correlations. The width of the edges indicates the strength of the connection. Some nodes display no connections with each other. Node colors indicate the community to which each node belongs.

## 4. Results

A total of 304 subjects were enrolled in the study. Of those, 12.5% (N = 38) dropped out of the study, and 4.9% (N = 15) died during the two-year follow up period. The drop-out reasons are summarized in Table S1. A description of sample characteristics at baseline is reported in Table 1.

### 4.1. Symptom Data Reduction 

Using state-level data of anxiety, depression and somatic symptoms, we identified four symptom complexes of state-like psychopathology, namely “Somatic”, “Depression”, “Agitation”, and “Anxiety”. Variable loadings for each network community are reported in the Supplementary Material (Table S2). 

### 4.2. Risk Factors for MDE at Follow Up

We compared baseline individuals on the basis of the occurrence of MDE at follow-up. Compared with those who did not go on to develop depression, those with depression were more likely to be female (29% with MDE, 17% without MDE), widowed (14% with MDE, 4.7% without MDE) and living alone (26% with MDE, 14% without MDE). They also displayed higher depression and psychopathological scores at baseline as well as higher scores for the following traits: Type D personality, novelty-seeking, harm avoidance, self-directedness, cooperativeness and difficulty identifying feelings. The sample baseline characteristics divided according to the occurrence of MDE during the follow-up period are shown in Table 2. 

Then, we compared the networks of state-level baseline symptoms across subjects with and without MDE at follow-up. 

#### 4.2.1. Network of the Baseline State Measures in the Sample Divided According to the Occurrence of MDE 

**Figure 1:**
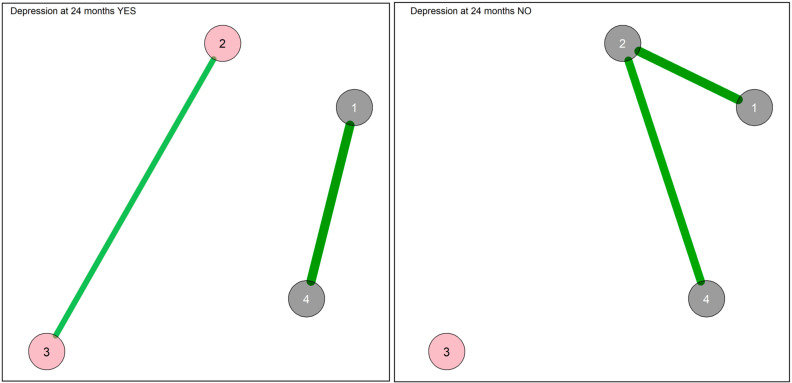
Network of symptoms with Bayesian estimation and walktrap community detection in the subgroups with and without MDE at 24 months. 1: Depression; 2: Somatic; 3: Agitation; 4: Anxiety. Each node corresponds to a symptom cluster identified in the previous symptom reduction. Nodes are connected with lines (edges) that represent the strength of association between nodes. Green edges suggest a positive association, whereas red edges suggest a negative association. The thickness of the edge represents the edge’s weight, which is an indication of the strength of the association. The thicker the edge, the greater the weight and the stronger the association. In this network, all edges connecting the nodes are green (positive correlation), with their width indicating the strength of their connections. Node colors indicate the community of the nodes. In the MDE YES network: pink for agitation and somatic symptoms and grey for depression and anxiety. In the MDE NO network: pink for agitation and grey for depression, anxiety and somatic symptoms.

The state-like symptom networks with Bayesian estimation in those that developed or did not develop an MDE during the follow-up period are displayed in Figure 1.

The two subgroups displayed different networks in terms of both connections and communities. The WAMs of the networks are reported in Table S7. In the network of those with MDE, two communities were identified, namely that of depression and anxiety and that of agitation/somatic symptoms, which were not connected to each other. Depression and anxiety displayed the highest positive connection between each other. In the network of those without MDE, the two communities were the same as in the whole sample, that is, that of agitation and depression and that of anxiety and somatic symptoms. Positive connections were observed between depression and somatic symptoms and between somatic symptoms and anxiety. The somatic node displayed the highest closeness of centrality, strength and expected influence, followed by those of depression and anxiety (Table S8). No significant differences were observed between the two groups in network comparison analyses.

#### 4.2.2. Network of the Trait Measures in the Sample Divided According to the Occurrence of MDE

**Figure 2:**
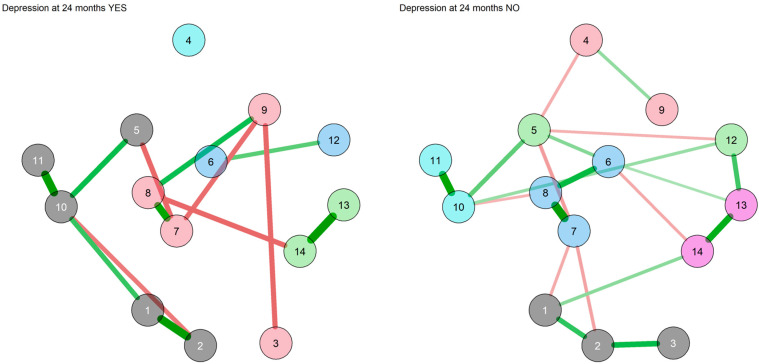
Network of trait measures with Bayesian estimation and walktrap community detection in the subgroups with and without MDE at 24 months. 1: TAS-20 Difficulty identifying feelings; 2: TAS-20 Difficulty describing feelings; 3: TAS-20 Externally oriented thinking; 4: TCI Novelty-seeking; 5: TCI Harm avoidance; 6: TCI Reward dependence; 7: TCI Self-directedness; 8: TCI Cooperativeness; 9: TCI Self-transcendence; 10: DS-14 Negative affectivity; 11: DS-14 Social inhibition; 12: DSQ-40 Mature defenses; 13: DSQ-40 Neurotic defenses; 14: DSQ-40 Immature defenses. Each node corresponds to a symptom cluster identified in the previous symptom reduction. Nodes are connected with lines (edges) that represent the strength of association between nodes. Green edges suggest a positive association, whereas red edges suggest a negative association. The thickness of the edge represents the edge’s weight, which is an indication of the strength of the association. The thicker the edge, the greater the weight and the stronger the association. In this network, all edges connecting the nodes are green (positive correlation), with their width indicating the strength of their connections. Node colors indicate the community of the nodes. In the MDE YES network: grey for inhibition, green for immature defenses, pink for TCI Characters, light blue for reward dependence and mature defenses and aquamarine for novelty-seeking. In the MDE NO network: pink for novelty-seeking and self-transcendence, grey for the TAS, green for harm avoidance and mature defenses, purple for immature defenses, light blue for TCI Reward dependence and characters and aquamarine for DS-14.

The trait-like measure networks with Bayesian estimation in the subgroups with and without MDE at follow-up are reported in Figure 2.

The two subgroups displayed different networks in terms of both connections and communities. In the network of those with MDE at follow-up, five communities were identified, namely inhibition (including the DS-14, the TAS DIF, the TAS DDF and the TCI Harm avoidance nodes), immature defenses (including the DSQ-40 Neurotic and Immature defenses nodes), TCI Characters (including the TCI Self-transcendence, Cooperativeness and Self-directedness nodes and the TAS EOT node), reward dependence and mature defenses (including the TCI Reward dependence node and the DSQ-40 Mature defenses nodes), and novelty-seeking (including only the TCI Novelty-seeking node). Two communities (the novelty-seeking and the reward dependence and mature defenses communities) did not display any connections with other communities. The network WAM and centrality measures are displayed in Tables S9 and S10. The strongest connections were those between the DSQ-40 Immature and Neurotic defenses nodes, followed by those between the TAS DIF and DDF nodes and between the DS-14 subscale nodes. The TCI Self-direction node displayed the greatest closeness centrality, whereas the node for DS-14 Negative affectivity displayed the highest strength. In the network of those without MDE at follow-up, six communities were identified, all of which were connected to at least one different community: novelty-seeking and self-transcendence (comprising the TCI Novelty-seeking and TCI Self-transcendence nodes), TAS (comprising the three TAS subscale nodes), harm avoidance and mature defenses (including the TCI Harm avoidance and the DSQ-40 Mature defenses nodes), immature defenses (including the DSQ-40 Neurotic and Immature defenses nodes), TCI Reward dependence and characters (including the TCI Reward dependence, Cooperativeness and Self-directedness nodes) and DS-14 (including the nodes of the DS-14 subscales Social inhibition and Negative affectivity). The network WAM and centrality measures are displayed in Tables S11 and S12. The strongest connections were those between the TCI Self-directedness and Cooperativeness nodes, followed by the connection between the nodes of the DS-14 subscales and that between the DSQ-40 Immature and DSQ-40 Neurotic defenses nodes. The TCI Harm avoidance node displayed the greatest closeness centrality, whereas the TCI Self-directedness node displayed the highest strength.

**Figure 3:**
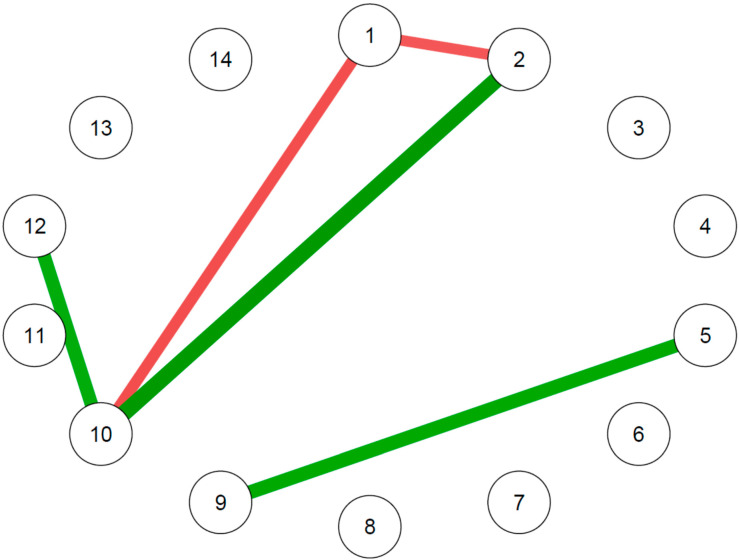
Differences of personality networks between those with and without MDE at follow-up. 1: TAS-20 Difficulty identifying feelings; 2: TAS-20 Difficulty describing feelings; 3: TAS-20 Externally oriented thinking; 4: TCI Novelty-seeking; 5: TCI Harm avoidance; 6: TCI Reward dependence; 7: TCI Self-directedness; 8: TCI Cooperativeness; 9: TCI Self-transcendence; 10: DS-14 Negative affectivity; 11: DS-14 Social inhibition; 12: DSQ-40 Mature defenses; 13: DSQ-40 Neurotic defenses; 14: DSQ-40 Immature defenses. Each node corresponds to a trait-like feature. The edges connecting the nodes indicate the main edge differences between the network of those with MDE and those without MDE, with green edges indicating positive differences and red edges indicating negative differences.

The trait-like features network comparison between those that developed or did not develop an MDE during the follow-up displayed differences in five edges (Figure 3).

Comparing the networks of trait measures between those with MDE and those without, some differences were identified. In particular, they differed in the edges connecting the TAS DIF and TAS DDF subscale nodes, the edges connecting the TAS DIF and the DS-14 Negative affectivity nodes, the TAS DDF and the DS-14 Negative affectivity nodes, the DS-14 Negative affectivity and DSQ-40 Mature defenses nodes and the TCI Harm avoidance and TCI Self-transcendence nodes. The highest difference was observed in the edge connecting the TAS DDF and the DS-14 Negative affectivity nodes, followed by the edge connecting the TCI Harm avoidance and Self-transcendence nodes and the edge connecting the DS-14 Negative affectivity and the DSQ-40 Mature defenses nodes (Table S13).

### 4.3. Analyses of the Sample Divided According to the Occurrence of MACE

The sample baseline characteristics divided according to the occurrence of MACE during the follow-up period are shown in Table 3. Patients that developed one or more MACE were older and displayed lower reward dependence personality traits. No other significant differences were observed. 

#### 4.3.1. Network of the State Measures in the Sample Divided According to the Occurrence of MACE

**Figure 4:**
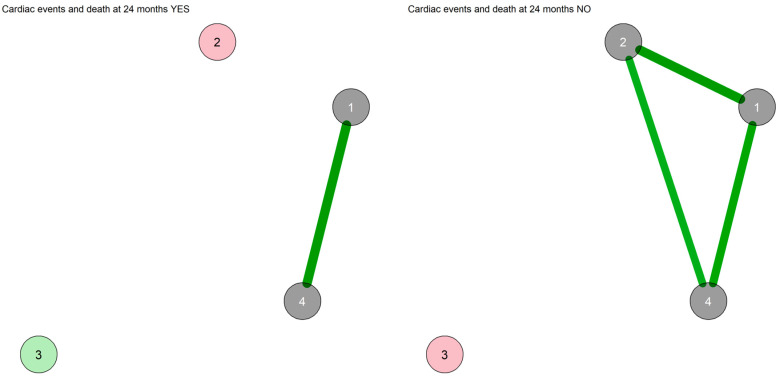
Network of symptoms with Bayesian estimation and walktrap community detection in the subgroups with and without MACE at 24 months. 1: Depression; 2: Somatic; 3: Agitation; 4: Anxiety. Each node corresponds to a symptom cluster identified in the previous symptom reduction. Nodes are connected with lines (edges) that represent the strengths of associations between nodes. Green edges suggest positive associations, whereas red edges suggest negative associations. The thickness of the edge represents the edge’s weight, which is an indication of the strength of the association. The thicker the edge, the greater the weight and the stronger the association. In this network, all edges connecting the nodes are green (positive correlation), and their widths indicate the strength of their connections. Node colors indicate the community of the nodes. In the MACE YES network, three communities were identified: somatic symptoms, depression and anxiety, and agitation. In the MACE NO network, two communities were identified: pink for agitation, and grey for somatic symptoms, depression and anxiety. All edges connecting the nodes are green (positive correlation), and their widths indicate the strength of their connections.

The state-like symptom networks with Bayesian estimation in those with and without the occurrence of one or more MACE during the follow-up are displayed in Figure 4.

The network of those with MACE at follow-up displayed three communities: somatic symptoms, depression and anxiety, and agitation. In the network of those without MACE at follow-up, two separate communities were identified (the same as in the whole sample): one with agitation only, and the other including somatic symptoms, depression and anxiety. The WAMs of the networks are reported in Table S14. In the network of those without MACE, somatic symptoms and depression displayed the strongest connection; the same connection was also present in the network of those with MACE at follow-up. The centrality measures of the networks are displayed in Table S15. In the network of those without MACE at follow-up, somatic symptoms displayed the highest closeness centrality, strength and expected influence, followed by depression and anxiety. No significant differences were observed between the two groups in network comparison analyses. When adjusting the analyses for age, no difference was observed.

#### 4.3.2. Network of the Trait Measures in the Sample Divided According to the Occurrence of MACE

**Figure 5:**
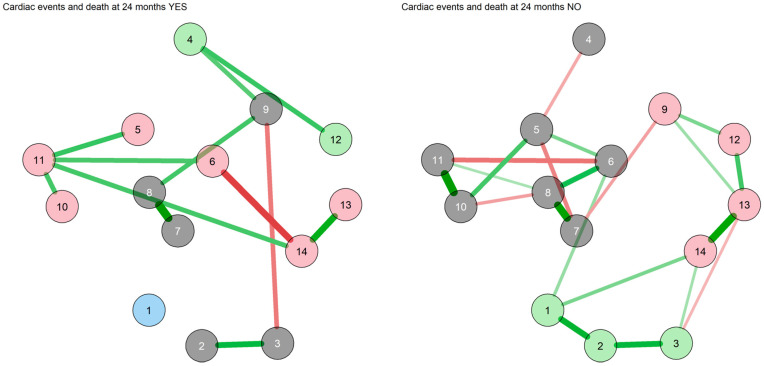
Network of trait measures with Bayesian estimation and walktrap community detection in the subgroups with and without MACE at follow-up. 1: TAS-20 Difficulty identifying feelings; 2: TAS-20 Difficulty describing feelings; 3: TAS-20 Externally oriented thinking; 4: TCI Novelty-seeking; 5: TCI Harm avoidance; 6: TCI Reward dependence; 7: TCI Self-directedness; 8: TCI Cooperativeness; 9: TCI Self-transcendence; 10: DS-14 Negative affectivity; 11: DS-14 Social inhibition; 12: DSQ-40 Mature defenses; 13: DSQ-40 Neurotic defenses; 14: DSQ-40 Immature defenses. Each node corresponds to a symptom cluster identified in the previous symptom reduction. Nodes are connected with lines (edges) that represent the strength of association between nodes. Green edges suggest positive associations, whereas red edges suggest negative associations. The thickness of the edge represents the edge’s weight, which is an indication of the strength of the association. The thicker the edge, the greater the weight and the stronger the association. In this network, all edges connecting the nodes are green (positive correlation), and their widths indicate the strength of their connections. Node colors indicate the community of the nodes. In the MACE YES network: grey for TCI characters, TAS DDF and TAS EOT; pink for immature defenses, harm avoidance and reward dependence and the DS-14; green for mature defenses and novelty-seeking; light blue for TAS DIF. In the MACE NO network: pink for defenses and the TCI Self-transcendence; green for the TAS; grey for the TCI personality traits and the DS-14.

The trait-like feature networks with Bayesian estimation in those with and without the occurrence of a MACE during the follow-up are displayed in Figure 5.

In the network of those with MACE at follow-up, four communities were identified: one including the TCI characters and TAS DDF and EOT nodes; one with the immature defenses, harm avoidance and reward dependence nodes and the DS-14; one with the mature defenses and novelty-seeking nodes; one comprising the TAS DIF node only with no connections to other communities. The WAM and the centrality measures of the network are displayed in Tables S16 and S17, respectively. The edges with the highest weight were those between the TCI Self-directedness and TCI Cooperativeness nodes, followed by the edge between the immature and neurotic defenses nodes and the negative connection between the TCI Reward dependence and DSQ-40 Immature defenses nodes. The TCI Self-directedness node displayed the highest closeness centrality, and the DS-14 Social inhibition node displayed the greatest strength.

In the network of those without MACE at follow-up, three communities were identified: all defense nodes and the TCI Self-transcendence node; the TAS subscales nodes; the TCI personality traits and the DS-14 nodes. The WAM and the centrality measures of the network are displayed in Tables S18 and S19 respectively. The strongest connections were those between the DS-14 subscale nodes, followed by the edge between the TCI Self-directedness and Cooperativeness nodes and the connection between the DSQ-40 Immature and Neurotic defenses nodes. The TCI Reward dependence node displayed the highest closeness centrality, whereas the TCI cooperativeness node displayed the highest strength. 

When adjusting the analyses for age, we observed difficulties in estimating the network because a non-positive definite matrix was obtained for the group with MACE at follow-up.

**Figure 6:**
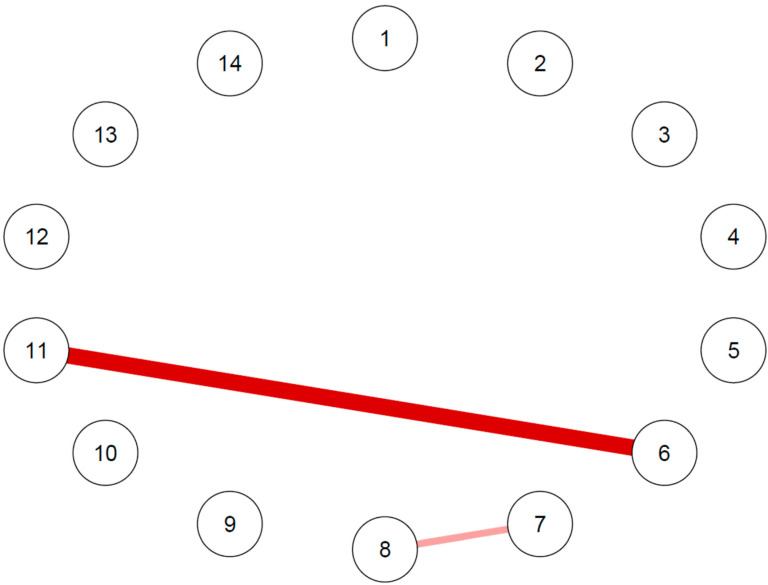
Graphical representation of edge differences in the trait-like features network with Bayesian estimation between those with and without MACE at follow-up. 1: TAS-20 Difficulty identifying feelings; 2: TAS-20 Difficulty describing feelings; 3: TAS-20 Externally oriented thinking; 4: TCI Novelty-seeking; 5: TCI Harm avoidance; 6: TCI Reward dependence; 7: TCI Self-directedness; 8: TCI Cooperativeness; 9: TCI Self-transcendence; 10: DS-14 Negative affectivity; 11: DS-14 Social inhibition; 12: DSQ-40 Mature defenses; 13: DSQ-40 Neurotic defenses; 14: DSQ-40 Immature defenses. Each node corresponds to a trait-like feature. The edges connecting the nodes indicate the main edge differences between the network of those with MACE and those without MACE. All edges are red, indicating negative differences.

The trait-features network comparison between those with and without MACE during the follow-up displayed differences in two edges (Figure 6).

In particular, the greatest difference was observed in the edge between the TCI Reward dependence and DS-14 Social inhibition nodes, whereas another minor difference was observed in the edge connecting the TCI Self-directedness and TCI Cooperativeness nodes (Table S20).

## 5. Discussion

We examined whether differences in symptoms and personality predicted the onset of depression or cardiac recurrences in patients with acute coronary syndrome and no previous history of depression. We found that stable personality features, less so transient symptoms, predicted the onset of depression and cardiac recurrence within two years. In particular, those with recurrent MDE displayed higher levels of depression, Type D personality traits and alexithymia as well as stronger associations between alexithymia and negative affectivity. 

### 5.1. Factors Associated with Depression at Follow-Up

In individuals after their first ACS and with no history of depression, networks of trait-like characteristics, in particular the connections between alexithymia, defense styles and personality traits such as negative affectivity, harm avoidance and self-transcendence, differentiated those who developed a depressive episode within a two-year follow-up period from those who did not. Alexithymia is associated with depression [66] and worsens depression and anxiety in individuals with a history of coronary heart disease [67]. In this study, individuals who developed an MDE at follow-up displayed higher difficulty in identifying feelings and stronger relationships between difficulty in describing and difficulty in identifying feelings, which are the TAS-20 items most associated with depression [66]. Moreover, they displayed stronger connections between difficulty in identifying feelings and negative affectivity as well as higher scores in both Type D personality traits, confirming the role of Type D personality as a risk factor for depression [68].

Those without MDE at follow-up instead displayed a stronger association between difficulty in describing feelings, negative affectivity and a mature defense style; defense mechanisms could play a mediating role between alexithymia and depression because impairments in emotion recognition and expression could lead to the development of poor emotion regulation strategies and maladaptive reactions to environmental stressors [69]. 

Significantly higher harm avoidance and self-directedness traits were observed in individuals with MDE at follow-up as well as a lower association between harm avoidance and self-transcendence than those without MDE at follow-up. High harm avoidance and low self-directedness traits confer vulnerability to depression [70,71]. The positive relationship between harm avoidance and self-transcendence in individuals without MDE at follow-up could be an indicator of that shift, protecting individuals from depression. Moreover, in the baseline personality network, harm avoidance is only indirectly related to self-transcendence via a negative relation to novelty-seeking, which in turn is positively associated with self-transcendence, with which it constitutes a community. In the framework of positive psychology, Wong perceived self-transcendence as a fundamental part of healing and well-being [72] that permits shifting focus from oneself to others, especially in the face of adversity [73]. Lower self-transcendence characteristics are also associated with depression [74,75], although in our sample, this trait did not display a significant difference between the two groups. 

State-like symptom network comparisons did not yield any significant differences between the MDE and non-MDE groups, although the baseline symptoms network displayed different communities and connections. Individuals with MDE at follow-up, in fact, showed a strong association between depression and anxiety at baseline as well as an association between somatic symptoms and agitation. In contrast, individuals without MDE at follow-up displayed a positive association between somatic symptoms and depression and anxiety. According to Ormel and de Jonge [76], depression in cardiovascular diseases could be exemplified by two types of prototypical depression: a cognitive/affective subtype, characterized by both specific coping styles that increase vulnerability to environmental stresses and specific personality traits such as neuroticism, which is paired together with the consequences of cardiovascular events; a somatic/affective subtype, characterized by alterations in inflammatory and neuroendocrine systems and a clinical picture mainly consisting of somatic symptoms. The two communities identified in the network analysis could represent these two different depression subtypes associated with cardiovascular diseases.

Interestingly, we previously showed that both the Type D personality, specifically the negative affectivity dimension [40,41,42], and alexithymia, specifically the difficulties in identifying and describing feelings [36,37,38,39], often overlap with subthreshold depressive symptoms. We cannot, hence, rule out completely that even in our cohort baseline, subthreshold depressive symptoms, especially anhedonic ones [35], may predict the onset of a full-blown depressive episode in the following two years, which would be in line with other longitudinal studies [77].

Eventually, depressive disorders affect up to one-third of the elderly population [78] and are associated with increased morbidity and mortality risks, including those related to cardiovascular diseases [79]. Thus, it should be of concern to identify and control depression in old age. Individuals’ trait-like features should therefore be considered when assessing the risk of late-life depression.

### 5.2. Factors Associated with Cardiovascular Events at Follow-Up

The development of a MACE at follow-up was associated with a positive connection between reward dependence and social inhibition and between self-directedness and cooperativeness. These results confirm the notion that temperament features and Type D personality characteristics represent risk factors for cardiovascular disease. However, in our sample, those with MACE at follow-up displayed significantly lower reward dependency. This contrasts with the literature on temperament features and cardiovascular risk: higher reward dependence, together with novelty-seeking and lower harm avoidance, are risk factors for atherosclerosis with an impact comparable to that of traditional risk factors for coronary heart disease [80]. Subjects with coronary heart disease display higher reward dependence compared to healthy controls, which is also negatively correlated with the ejection fraction [81]. The association between reward dependence and social inhibition we observed in this study could be explained as reward sensitivity and a need to avoid errors in social contexts, which is associated with behavioral inhibition and social anxiety [82], leading to high distress. In fact, the Type D personality is a risk factor for both depression and cardiovascular diseases, possibly increasing stress-related hormones [83].

Patients who developed one or more MACE at follow-up also displayed a slightly stronger association between self-directedness and cooperativeness. These two personality traits are thought to be dependent on good “theory of mind” acquisition, being associated with adequate self-experiences and experiences with others, and they proved to be deficient in individuals with neurodevelopmental disorders as well as with alcohol and substance use disorders [84,85]. 

No significant difference was observed in the network comparison of state-like symptoms between those with and without one or more MACE at follow-up; however, the baseline symptoms network displayed different communities and connections. In contrast to a meta-analysis that observed that only somatic/affective symptoms and not cognitive/affective ones were associated with cardiovascular prognoses in patients with depression [86], no significant difference was found in this study in the network comparison of state-like symptoms between those with and without an MDE at follow-up. 

It is also possible that baseline state-like features were not specifically associated with MACE in this specific analysis, as the role of baseline depressive or anxious symptoms have a differential impact on the cardiac outcome based on the conditional development of depression [87]. It was, in fact, suggested that higher levels of anxiety were associated with worse cardiac outcomes in those that develop depressive episodes due to the cardiotoxic effect of higher adrenergic stimulation, but, conversely, it might exert a protective effect in those that remain euthymic over the follow-up period [88]. Increased levels of anxiety may, hence, reflect the worry after the first diagnosis of ACS, which could induce greater compliance with a medical prescription and promote adaptive coping strategies [15] and healthy behaviors [16,17].

These results need to be considered in light of the limitations of this study. First, we recruited individuals at their first cardiovascular event to compare those with and without a depressive episode or a second cardiac event/death. Thus, we did not calculate a sample size beforehand for the groups to be compared. The different sample sizes between groups could thereby limit the strength of the results. Second, the small sample size prevented us from differentiating and comparing individuals according to different types of acute cardiac events. Third, because the study focused on the individual state- and trait-like features, biological biomarker levels, such as those of troponins and CK-MB, and variables related to pharmacotherapies were not collected and included in the analyses, although biological parameters could be implicated in the pathogenesis of MACE and MDEs at follow-up. 

The results of the present study, however, indicate the need to implement psychiatric evaluation in the clinical care of individuals with ACS. The assessment of personality characteristics should be included in routine clinical practice in order to identify those at higher risk for the development of a depressive disorder and further cardiovascular events. Vulnerable individuals should therefore be referred to appropriate psychological and psychiatric interventions with the aim of improving coping strategies to mitigate risk, improve adaptation to the disease and eventually increase quality of life.

## 6. Conclusions

Depressive symptoms and episodes are frequent in individuals who have suffered a cardiovascular event. The present study observed that trait-like features, in particular alexithymia and personality traits such as negative affectivity, harm avoidance and self-transcendence, and especially their reciprocal relationships, are more evident in individuals who develop a depressive episode at follow-up; thus, they possibly represent predisposing factors for a subsequent depressive episode. The assessment of personality features in patients with a cardiovascular event could thereby aid in the identification of those with a higher risk for depression; adequate specialist referral and interventions could reduce risk and increase the quality of life of patients suffering from a cardiovascular disease.

## Figures and Tables

**Table 1:** Sample characteristics. ^1^ Mean (SD); *n* (%). HADS: Hospital Anxiety and Depression Scale; PRIME-MD: Primary Care Evaluation of Mental Disorder; PHQ: Patient Health Questionnaire; TCI: Temperament and Character Inventory; DS-14: Type D Personality Scale; TAS-20: Toronto Alexithymia Scale, 20-item; DSQ-40: Defense Style Questionnaire.

Characteristic	N = 304 ^1^
Age	61.43 (10.98)
Gender	
Female	59 (19%)
Male	245 (81%)
Education	10.13 (3.75)
Marital status	
Single	36 (12%)
In a relationship	223 (73%)
Divorced	24 (7.9%)
Widowed	21 (6.9%)
Occupation	
Unemployed	8 (2.6%)
Student	0 (0%)
Housewife	16 (5.3%)
Employed	130 (43%)
Retired	150 (49%)
Living	
Alone	52 (17%)
Not alone	252 (83%)
Family support	
Problematic	3 (1.0%)
Fair	36 (12%)
Good	265 (87%)
Smoking	93 (36%)
Past antidepressants	1 (0.3%)
HADS Anxiety	9.12 (5.13)
HADS Depression	7.11 (4.22)
HADS total score	16.24 (8.81)
PRIME MD total score	2.87 (3.08)
PRIME MD PHQ total score	15.07 (6.66)
TAS-20 DIF	15.07 (5.73)
TAS-20 DDF	13.02 (4.23)
TAS-20 EOT	21.22 (4.30)
TCI Novelty-seeking (NS)	17.58 (4.94)
TCI Harm avoidance (HA)	15.91 (5.59)
TCI Reward dependence (RD)	14.05 (3.21)
TCI Self-directedness (SD)	29.50 (7.03)
TCI Cooperativeness (C)	29.69 (5.74)
TCI Self-transcendence (ST)	14.44 (5.69)
DS-14 Negative affectivity (NA)	8.98 (6.00)
DS-14 Social inhibition (SI)	10.21 (4.98)
DSQ-40 Mature	5.00 (1.18)
DSQ-40 Neurotic	4.14 (1.39)
DSQ-40 Immature	3.97 (1.14)

**Table 2:** Sample baseline characteristics according to the occurrence of MDE during the follow-up period. ^1^ Mean (SD); n (%). ^2^ Wilcoxon rank-sum test; Pearson’s chi-squared test; Fisher’s exact test. HADS: Hospital Anxiety and Depression Scale; PRIME-MD: Primary Care Evaluation of Mental Disorder; PHQ: Patient Health Questionnaire; TCI: Temperament and Character Inventory; DS-14: Type-D Personality Scale; TAS-20: Toronto Alexithymia Scale, 20-item; DSQ-40: Defense Style Questionnaire. * *p* < 0.05.

Characteristic	No, N = 235 ^1^	Yes, N = 69 ^1^	*p*-Value ^2^
Age	61.16 (11.02)	62.33 (10.86)	0.359
Gender			0.022 *
Female	39 (17%)	20 (29%)	
Male	196 (83%)	49 (71%)	
Education	10.16 (3.74)	10.04 (3.79)	0.878
Marital status			0.038 *
Single	29 (12%)	7 (10%)	
In a relationship	178 (76%)	45 (65%)	
Divorced	17 (7.2%)	7 (10%)	
Widowed	11 (4.7%)	10 (14%)	
Occupation			0.159
Unemployed	6 (2.6%)	2 (2.9%)	
Student	0 (0%)	0 (0%)	
Housewife	9 (3.8%)	7 (10%)	
Employed	105 (45%)	25 (36%)	
Retired	115 (49%)	35 (51%)	
Living			0.024 *
Alone	34 (14%)	18 (26%)	
Not alone	201 (86%)	51 (74%)	
Family support			0.030 *
Problematic	2 (0.9%)	1 (1.4%)	
Fair	22 (9.4%)	14 (20%)	
Good	211 (90%)	54 (78%)	
Smoking	73 (36%)	20 (36%)	0.995
Past depression	0 (0%)	0 (0%)	>0.999
Past antidepressants	0 (0%)	1 (1.4%)	0.227
HADS Anxiety	9.03 (5.31)	9.46 (4.48)	0.999
HADS Depression	6.75 (4.21)	8.35 (4.04)	0.003 *
HADS total score	15.77 (9.05)	17.81 (7.81)	0.164
PRIME MD total score	2.19 (2.13)	5.19 (4.43)	<0.001 *
PRIME MD PHQ total score	14.54 (6.45)	17.00 (7.09)	0.004 *
TAS-20 DIF	14.53 (5.55)	16.90 (6.01)	0.004 *
TAS-20 DDF	12.87 (4.24)	13.55 (4.21)	0.159
TAS-20 EOT	21.18 (4.35)	21.38 (4.18)	0.820
TCI Novelty-seeking (NS)	17.16 (4.70)	18.98 (5.47)	0.013 *
TCI Harm avoidance (HA)	15.11 (5.53)	18.64 (4.92)	<0.001 *
TCI Reward dependence (RD)	13.97 (3.24)	14.34 (3.10)	0.640
TCI Self-directedness (SD)	30.30 (6.92)	26.80 (6.77)	<0.001 *
TCI Cooperativeness (C)	30.15 (5.59)	28.13 (6.00)	0.019 *
TCI Self-transcendence (ST)	14.20 (5.60)	15.25 (5.94)	0.298
DS-14 Negative affectivity (NA)	8.00 (5.33)	12.33 (6.91)	<0.001 *
DS-14 Social inhibition (SI)	9.60 (4.62)	12.30 (5.62)	<0.001 *
DSQ-40 Mature	5.00 (1.18)	5.00 (1.18)	0.784
DSQ-40 Neurotic	4.09 (1.41)	4.31 (1.32)	0.578
DSQ-40 Immature	3.90 (1.15)	4.21 (1.09)	0.062

**Table 3:** Comparing groups with and without MACE at 24-month follow-up. ^1^ Mean (SD); n (%). ^2^ Wilcoxon rank-sum test; Pearson’s chi-squared test; Fisher’s exact test. HADS: Hospital Anxiety and Depression Scale; PRIME-MD: Primary Care Evaluation of Mental Disorder; PHQ: Patient Health Questionnaire; TCI: Temperament and Character Inventory; DS-14: Type-D Personality Scale; TAS-20: Toronto Alexithymia Scale, 20-item; DSQ-40: Defense Style Questionnaire.

Characteristic	No, N = 247 ^1^	Yes, N = 57 ^1^	*p*-Value ^2^
Age	60.53 (11.19)	65.32 (9.09)	0.002
Gender			0.443
Female	50 (20%)	9 (16%)	
Male	197 (80%)	48 (84%)	
Education	10.26 (3.70)	9.56 (3.92)	0.172
Marital status			0.146
Single	33 (13%)	3 (5.3%)	
In a relationship	176 (71%)	47 (82%)	
Divorced	22 (8.9%)	2 (3.5%)	
Widowed	16 (6.5%)	5 (8.8%)	
Occupation			0.247
Unemployed	8 (3.2%)	0 (0%)	
Student	0 (0%)	0 (0%)	
Housewife	12 (4.9%)	4 (7.0%)	
Employed	110 (45%)	20 (35%)	
Retired	117 (47%)	33 (58%)	
Living			0.770
Alone	43 (17%)	9 (16%)	
Not alone	204 (83%)	48 (84%)	
Family support			0.906
Problematic	3 (1.2%)	0 (0%)	
Fair	30 (12%)	6 (11%)	
Good	214 (87%)	51 (89%)	
Smoking	76 (37%)	17 (34%)	0.703
Past depression	0 (0%)	0 (0%)	>0.999
Past antidepressants	1 (0.4%)	0 (0%)	>0.999
HADS Anxiety	9.04 (5.17)	9.49 (4.94)	0.640
HADS Depression	7.07 (4.26)	7.30 (4.07)	0.707
HADS total score	16.11 (8.93)	16.79 (8.33)	0.603
PRIME MD total score	2.80 (2.84)	3.16 (3.98)	0.521
PRIME MD PHQ total score	15.13 (6.80)	14.82 (6.09)	0.808
TAS-20 DIF	15.14 (5.76)	14.77 (5.68)	0.702
TAS-20 DDF	13.11 (4.31)	12.67 (3.88)	0.511
TAS-20 EOT	21.05 (4.33)	21.98 (4.13)	0.235
TCI Novelty-seeking (NS)	17.65 (4.81)	17.28 (5.46)	0.981
TCI Harm avoidance (HA)	16.10 (5.72)	15.15 (5.02)	0.326
TCI Reward dependence (RD)	14.33 (3.00)	12.92 (3.78)	0.011
TCI Self-directedness (SD)	29.37 (6.98)	30.02 (7.27)	0.344
TCI Cooperativeness (C)	29.98 (5.51)	28.53 (6.53)	0.189
TCI Self-transcendence (ST)	14.64 (5.53)	13.60 (6.29)	0.243
DS-14 Negative affectivity (NA)	8.90 (6.09)	9.33 (5.64)	0.515
DS-14 Social inhibition (SI)	10.09 (4.92)	10.74 (5.27)	0.259
DSQ-40 Mature	5.00 (1.18)	5.00 (1.17)	0.973
DSQ-40 Neurotic	4.14 (1.33)	4.14 (1.63)	0.962
DSQ-40 Immature	3.95 (1.14)	4.02 (1.16)	0.594

## Data Availability

The data presented in this study are available on request from the corresponding author. The data are not publicly available due to privacy reasons.

## Supplementary Materials

The following supporting information can be downloaded at: https://www.mdpi.com/article/10.3390/diagnostics13050915/s1, Figure S1. Network of state-like measures with Bayesian estimation and walktrap community detection in the whole sample at baseline; Figure S2. Network of trait-like measures with Bayesian estimation and walktrap community detection in the whole sample at baseline; Table S1. State-like symptoms loadings for each network community; Table S2. Weighted Adjacency Matrix of the state-like symptoms network with Bayesian estimation in the whole sample; Table S3. Centrality measures of the state-like symptoms network with Bayesian estimation in the whole sample; Table S4. Weighted Adjacency Matrix of the trait-like features network with Bayesian estimation in the whole sample; Table S5. Centrality measures of the state-like symptoms network with Bayesian estimation in the whole sample; Table S6. Weighted Adjacency Matrix of the state-like symptoms network with Bayesian estimation in those with and without MDE at follow-up; Table S7. Centrality measures of the state-like symptoms network with Bayesian estimation in those without MDE at follow-up; Table S8. Weighted Adjacency Matrix of the trait-like features network with Bayesian estimation in those with MDE at follow-up; Table S9. Centrality measures of the trait-like features network with Bayesian estimation in those with MDE at follow-up; Table S10. Weighted Adjacency Matrix of the trait-like features network with Bayesian estimation in those without MDE at follow-up; Table S11. Centrality measures of the trait-like features network with Bayesian estimation in those without MDE at follow-up; Table S12. Edge differences in the trait-like features network with Bayesian estimation between those with and without depression at follow-up; Table S13. Weighted Adjacency Matrix of the state-like symptoms network with Bayesian estimation in those without MACE at follow-up; Table S14. Centrality measures of the state-like symptoms network with Bayesian estimation in those without MACE at follow-up; Table S15. Weighted Adjacency Matrix of the trait-like features network with Bayesian estimation in those without MDE at follow-up; Table S16. Centrality measures of the trait-like features network with Bayesian estimation in those without MDE at follow-up; Table S17. Weighted Adjacency Matrix of the trait-like features network with Bayesian estimation in those without MDE at follow-up; Table S18. Centrality measures of the trait-like features network with Bayesian estimation in those without MDE at follow-up; Table S19. Edge differences in the trait-like features network with Bayesian estimation between those with and without MACE at follow-up.

## References

[1] Frost J., Rich R.L., Robbins C.W., Stevermer J.J., Chow R.T., Leon K.K., Bird M.D. (2019). Depression Following Acute Coronary Syndrome Events: Screening and Treatment Guidelines from the AAFP. Am. Fam. Phys..

[2] Lim G.B. (2014). Risk Factors: Depression Recognized as a Risk Factor in ACS. Nat. Rev. Cardiol..

[3] Alhurani A.S., Hamdan-Mansour A.M., Ahmad M.M., McKee G., O’donnell S., O’brien F., Mooney M., Saleh Z.T., Moser D.K. (2022). The Association of Persistent Symptoms of Depression and Anxiety with Recurrent Acute Coronary Syndrome Events: A Prospective Observational Study. Healthcare.

[4] Lichtman J.H., Froelicher E.S., Blumenthal J.A., Carney R.M., Doering L.V., Frasure-Smith N., Freedland K.E., Jaffe A.S., Leifheit-Limson E.C., Sheps D.S. (2014). Depression as a Risk Factor for Poor Prognosis among Patients with Acute Coronary Syndrome: Systematic Review and Recommendations: A Scientific Statement from the American Heart Association. Circulation.

[5] Smolderen K.G., Buchanan D.M., Gosch K., Whooley M., Chan P.S., Vaccarino V., Parashar S., Shah A.J., Ho P.M., Spertus J.A. (2017). Depression Treatment and 1-Year Mortality After Acute Myocardial Infarction: Insights From the TRIUMPH Registry (Translational Research Investigating Underlying Disparities in Acute Myocardial Infarction Patients’ Health Status). Circulation.

[6] Ossola P., Generali I., Schito G., De Panfilis C., Tonna M., Gerra M.L., Marchesi C. (2019). Temperament and Depression After a First Acute Coronary Syndrome. J. Nerv. Ment. Dis..

[7] Vaccarino V., Badimon L., Bremner J.D., Cenko E., Cubedo J., Dorobantu M., Duncker D.J., Koller A., Manfrini O., Milicic D. (2020). Depression and Coronary Heart Disease: 2018 Position Paper of the ESC Working Group on Coronary Pathophysiology and Microcirculation. Eur. Heart J..

[8] Mandarano P., Ossola P., Castiglioni P., Faini A., Marazzi P., Carsillo M., Rozzi S., Lazzeroni D. (2022). Heart Rate Fractality Disruption as a Footprint of Subthreshold Depressive Symptoms in a Healthy Population. Clin. Neuropsychiatry.

[9] Milaneschi Y., Kappelmann N., Ye Z., Lamers F., Moser S., Jones P.B., Burgess S., Penninx B.W.J.H., Khandaker G.M. (2021). Association of Inflammation with Depression and Anxiety: Evidence for Symptom-Specificity and Potential Causality from UK Biobank and NESDA Cohorts. Mol. Psychiatry.

[10] Hare D.L., Toukhsati S.R., Johansson P., Jaarsma T. (2014). Depression and Cardiovascular Disease: A Clinical Review. Eur. Heart J..

[11] Zhou Y., Huo Q., Du S., Shi X., Shi Q., Cui S., Feng C., Du X., Wang Y. (2022). Social Support and Self-Efficacy as Mediating Factors Affecting the Association Between Depression and Medication Adherence in Older Patients with Coronary Heart Disease: A Multiple Mediator Model with a Cross-Sectional Study. Patient Prefer. Adherence.

[12] Fan Y., Shen B.J., Tay H.Y. (2021). Depression, Anxiety, Perceived Stress, and Their Changes Predicted Medical Adherence over 9 Months among Patients with Coronary Heart Disease. Br. J. Health Psychol..

[13] Gehi A., Haas D., Pipkin S., Whooley M.A. (2005). Depression and Medication Adherence in Outpatients with Coronary Heart Disease: Findings from the Heart and Soul Study. Arch. Intern. Med..

[14] Suskin N.G., Huitema A.A., Hartley T., McKelvie R.S. (2021). Sex, Depression, and More in Cardiac Rehabilitation. Can. J. Cardiol..

[15] Messerli-Bürgy N., Molloy G.J., Poole L., Wikman A., Kaski J.C., Steptoe A. (2015). Psychological Coping and Recurrent Major Adverse Cardiac Events Following Acute Coronary Syndrome. Br. J. Psychiatry.

[16] Benyamini Y., Roziner I., Goldbourt U., Drory Y., Gerber Y. (2013). Depression and Anxiety Following Myocardial Infarction and Their Inverse Associations with Future Health Behaviors and Quality of Life. Ann. Behav. Med..

[17] Gale C.R., Čukić I., Batty G.D., McIntosh A.M., Weiss A., Deary I.J. (2017). When Is Higher Neuroticism Protective Against Death? Findings From UK Biobank. Psychol. Sci..

[18] Li X.-Q., Tang X.-R., Li L.-L. (2021). Antipsychotics Cardiotoxicity: What’s Known and What’s Next. WJP.

[19] Dickens C.M., Percival C., McGowan L., Douglas J., Tomenson B., Cotter L., Heagerty A., Creed F.H. (2004). The Risk Factors for Depression in First Myocardial Infarction Patients. Psychol. Med..

[20] Leong L.K., Zuhdi A.S.M., Hafidz M.I.A. (2021). Clinical Depression among Patients after Acute Coronary Syndrome: A Prospective Single-Tertiary Centre Analysis. Singap. Med. J..

[21] Doyle F., McGee H., Conroy R., Conradi H.J., Meijer A., Steeds R., Sato H., Stewart D.E., Parakh K., Carney R. (2015). Systematic Review and Individual Patient Data Meta-Analysis of Sex Differences in Depression and Prognosis in Persons With Myocardial Infarction: A MINDMAPS Study. Psychosom. Med..

[22] Carney R.M., Freedland K.E., Steinmeyer B., Blumenthal J.A., De Jonge P., Davidson K.W., Czajkowski S.M., Jaffe A.S. (2009). History of Depression and Survival after Acute Myocardial Infarction. Psychosom. Med..

[23] Frasure-Smith N., Lespérance F., Gravel G., Masson A., Juneau M., Talajic M., Bourassa M.G. (2000). Social Support, Depression, and Mortality during the First Year after Myocardial Infarction. Circulation.

[24] Denollet J., Sys S.U., Brutsaert D.L. (1995). Personality and Mortality after Myocardial Infarction. Psychosom. Med..

[25] De Fazio P., Caroleo M., Rizza P., Cerminara G., De Serio D., Indolfi C., Segura-García C. (2012). Specific Personality Traits and Coping Styles Predict Affective Symptoms in Early Post Acute Coronary Syndrome Inpatients. Int. J. Psychiatry Med..

[26] Romppel M., Herrmann-Lingen C., Vesper J.M., Grande G. (2012). Type D Personality and Persistence of Depressive Symptoms in a German Cohort of Cardiac Patients. J. Affect. Disord..

[27] Roberts S.B., Kendler K.S. (1999). Neuroticism and self-esteem as indices of the vulnerability to major depression in women. Psychol. Med..

[28] Parker G.B., Cvejic E., Vollmer-Conna U., McCraw S., Granville Smith I., Walsh W.F. (2019). Depression and Poor Outcome after an Acute Coronary Event: Clarification of Risk Periods and Mechanisms. Aust. N. Z. J. Psychiatry.

[29] Carless D., Douglas K., Fox K., McKenna J. (2006). An Alternative View of Psychological Well-Being in Cardiac Rehabilitation: Considering Temperament and Character. Eur. J. Cardiovasc. Nurs..

[30] Peters R.M., Lumley M.A. (2007). Relationship of Alexithymia to Cardiovascular Disease Risk Factors among African Americans. Compr. Psychiatry.

[31] Kojima M., Frasure-Smith N., Lesperance F. (2001). Alexithymia Following Myocardial Infarction Psychometric Properties and Correlates of the Toronto Alexithymia Scale. J. Psychosom. Res..

[32] 32. Honkalampi K., Hintikka J., Tanskanen A., Lehtonen J., Viinamäki H. (2000). Depression is strongly associated with alexithymia in the general population. J. Psychosom. Res..

[33] Epstein S., O’Brien E.J. (1985). The Person-Situation Debate in Historical and Current Perspective. Psychol. Bull..

[34] Steyer R., Schmitt M., Eid M. (1999). Latent State-Trait Theory and Research in Personality and Individual Differences. Eur. J. Personal..

[35] Ossola P., Paglia F., Pelosi A., De Panfilis C., Conte G., Tonna M., Ardissino D., Marchesi C. (2015). Risk Factors for Incident Depression in Patients at First Acute Coronary Syndrome. Psychiatry Res..

[36] Marchesi C., Ossola P., Scagnelli F., Mellini L., Tonna M., Ardissino D., De Panfilis C. (2015). The Role of Alexithymia in Predicting Incident Depression in Patients at First Acute Coronary Syndrome. Compr. Psychiatry.

[37] Marchesi C., Ossola P., Tonna M., De Panfilis C. (2014). The TAS-20 More Likely Measures Negative Affects Rather than Alexithymia Itself in Patients with Major Depression, Panic Disorder, Eating Disorders and Substance Use Disorders. Compr. Psychiatry.

[38] Ossola P., Gerra M.L., Beltrani M., Marchesi C. (2019). Alexithymia and Cardiac Outcome in Patients at First Acute Coronary Syndrome. Int.J. Behav. Med..

[39] De Panfilis C., Ossola P., Tonna M., Catania L., Marchesi C. (2015). Finding Words for Feelings: The Relationship between Personality Disorders and Alexithymia. Personal. Individ. Differ..

[40] Marchesi C., Ossola P., Scagnelli F., Paglia F., Aprile S., Monici A., Tonna M., Conte G., Masini F., De Panfilis C. (2014). Type D Personality in Never-Depressed Patients and the Development of Major and Minor Depression after Acute Coronary Syndrome. J. Affect. Disord..

[41] Marchesi C., Ossola P., Scagnelli F., Paglia F., Aprile S., Monici A., Tonna M., Conte G., Masini F., De Panfilis C. (2014). Type D Personality in Never Depressed Patients at Their First Acute Coronary Syndrome. Psychother. Psychosom..

[42] Ossola P., De Panfilis C., Tonna M., Ardissino D., Marchesi C. (2015). DS14 Is More Likely to Measure Depression Rather than a Personality Disposition in Patients with Acute Coronary Syndrome. Scand. J. Psychol..

[43] Borsboom D., Cramer A.O.J. (2013). Network Analysis: An Integrative Approach to the Structure of Psychopathology. Annu. Rev. Clin. Psychol..

[44] Costantini G., Epskamp S., Borsboom D., Perugini M., Mõttus R., Waldorp L.J., Cramer A.O.J. (2015). State of the ARt Personality Research: A Tutorial on Network Analysis of Personality Data in R. J. Res. Personal..

[45] Borsboom D. (2017). A Network Theory of Mental Disorders. World Psychiatry.

[46] Hamm C.W., Bassand J.P., Agewall S., Bax J., Boersma E., Bueno H., Caso P., Dudek D., Gielen S., Huber K. (2011). ESC Guidelines for the Management of Acute Coronary Syndromes in Patients Presenting without Persistent ST-Segment Elevation. Eur. Heart J..

[47] Van De Werf F., Bax J., Betriu A., Blomstrom-Lundqvist C., Crea F., Falk V., Filippatos G., Fox K., Huber K., Kastrati A. (2008). Management of Acute Myocardial Infarction in Patients Presenting with Persistent ST-Segment Elevation. Eur. Heart J..

[48] Folstein M.F., Folstein S.E., McHugh P.R. (1974). “Mini-Mental State”. A Practical Method for Grading the Cognitive State of Patients for the Clinician. J. Psychiatr. Res..

[49] APA Diagnostic and Statistical Manual of Mental Disorders (2000). Text Revision (DSM-IV-TR). Diagnostic and Statistical Manual of Mental Disorders.

[50] Eagle K.A., Lim M.J., Dabbous O.H., Pieper K.S., Goldberg R.J., Goodman S.G., Granger C.B., Steg P.G., Gore J.M., Flather M.D. (2004). A Validated Prediction Model for All Forms of Acute Coronary Syndrome Estimating the Risk of 6-Month Postdischarge Death in an International Registry. JAMA.

[51] Osler M., Mårtensson S., Wium-Andersen I.K., Prescott E., Andersen P.K., Jørgensen T.S.H., Carlsen K., Wium-Andersen M.K., Jørgensen M.B. (2016). Depression After First Hospital Admission for Acute Coronary Syndrome: A Study of Time of Onset and Impact on Survival. Am. J. Epidemiol..

[52] Panchal H.B., Ladia V., Desai S. (2013). A Meta-Analysis of Mortality and Major Adverse Cardiovascular and Cerebrovascular Events Following Transcatheter Aortic Valve Implantation Versus Surgical Aortic Valve Replacement for Severe Aortic Stenosis. Am. J. Cardiol..

[53] Moise N., Khodneva Y., Jannat-khah D.P., Richman J., Davidson K.W., Kronish I.M., Shaffer J., Safford M.M. (2018). Observational Study of the Differential Impact of Time-Varying Depressive Symptoms on All-Cause and Cause- Specific Mortality by Health Status in Community-Dwelling Adults: The REGARDS Study. BMJ Open.

[54] Patti G., Cannon C.P., Murphy S.A., Mega S., Pasceri V., Briguori C., Colombo A., Yun K.H., Jeong M.H., Kim J.S. (2011). Clinical Benefit of Statin Pretreatment in Patients Undergoing Percutaneous Coronary Intervention: A Collaborative Patient-Level Meta-Analysis of 13 Randomized Studies. Circulation.

[55] de Ron J., Robinaugh D.J., Fried E.I., Pedrelli P., Jain F.A., Mischoulon D., Epskamp S. (2022). Quantifying and Addressing the Impact of Measurement Error in Network Models. Behav. Res. Ther..

[56] Christensen A.P., Garrido L.E., Golino H. (2020). Unique Variable Analysis: A Network Psychometrics Method to Detect Local Dependence. PsyArXiv.

[57] Golino H.F., Demetriou A. (2017). Estimating the Dimensionality of Intelligence like Data Using Exploratory Graph Analysis. Intelligence.

[58] Belvederi Murri M., Grassi L., Caruso R., Nanni M.G., Zerbinati L., Andreas S., Ausín B., Canuto A., Härter M., Lopez M.M. (2022). Depressive Symptom Complexes of Community-Dwelling Older Adults: A Latent Network Model. Mol. Psychiatry.

[59] Golino H., Shi D., Garrido L.E., Christensen A., Nieto M.D. (2018). Investigating the Performance of Exploratory Graph Analysis and Traditional Techniques to Identify the Number of Latent Factors: A Simulation and Tutorial. PsyArXiv.

[60] Golino H., Christensen A.P., Golino H., Christensen A.P. EGAnet: Exploratory Graph Analysis: A Framework for Estimating the Number of Dimensions in Multivariate Data Using Network Psychometrics. https://cran.r-project.org/package=EGAnet.

[61] Christensen A.P., Golino H. (2021). On the Equivalency of Factor and Network Loadings. Behav. Res. Methods.

[62] Golino H.F., Epskamp S. (2017). Exploratory Graph Analysis: A New Approach for Estimating the Number of Dimensions in Psychological Research. PLoS ONE.

[63] Williams D.R., Rast P., Pericchi L.R., Mulder J. (2020). Comparing Gaussian Graphical Models With the Posterior Predictive Distribution and Bayesian Model Selection. Psychol. Methods.

[64] Williams D., Mulder J. (2020). BGGM: Bayesian Gaussian Graphical Models. J. Open Source Softw..

[65] Epskamp S., Cramer A.O.J., Waldorp L.J., Schmittmann V.D., Borsboom D. (2012). Qgraph: Network Visualizations of Relationships in Psychometric Data. J. Stat. Softw..

[66] Li S., Zhang B., Guo Y., Zhang J. (2015). The Association between Alexithymia as Assessed by the 20-Item Toronto Alexithymia Scale and Depression: A Meta-Analysis. Psychiatry Res..

[67] Nekouei Z.K., Doost H.T., Yousefy A., Manshaee G., Sadeghei M. (2014). The Relationship of Alexithymia with Anxiety-Depression-Stress, Quality of Life, and Social Support in Coronary Heart Disease (A Psychological Model). J. Educ. Health Promot..

[68] van Dooren F.E.P., Verhey F.R.J., Pouwer F., Schalkwijk C.G., Sep S.J.S., Stehouwer C.D.A., Henry R.M.A., Dagnelie P.C., Schaper N.C., van der Kallen C.J.H. (2016). Association of Type D Personality with Increased Vulnerability to Depression: Is There a Role for Inflammation or Endothelial Dysfunction?—The Maastricht Study. J. Affect. Disord..

[69] Lenzo V., Barberis N., Cannavò M., Filastro A., Verrastro V., Quattropani M.C. (2020). The Relationship between Alexithymia, Defense Mechanisms, Eating Disorders, Anxiety and Depression. Riv. Di Psichiatr..

[70] Mochcovitch M.D., Nardi A.E., Cardoso A. (2012). Temperament and Character Dimensions and Their Relationship to Major Depression and Panic Disorder. Rev. Bras. De Psiquiatr..

[71] Bajraktarov S., Gudeva-Nikovska D., ManuÅ¡eva N., Arsova S. (2017). Personality Characteristics as Predictive Factors for the Occurrence of Depressive Disorder. Open Access Maced. J. Med. Sci..

[72] Wong P.T.P., Batthyány A. (2016). Meaning-Seeking, Self-Transcendence, and Well-being. Logotherapy and Existential Analysis: Proceedings of the Viktor Frankl Institute Vienna.

[73] Wong P.T.P., Mayer C.-H., Arslan G. (2021). Editorial: COVID-19 and Existential Positive Psychology (PP2.0): The New Science of Self-Transcendence. Front. Psychol..

[74] Liu P., Wang X., Li D., Zhang R., Li H., Han J. (2021). The Benefits of Self-Transcendence: Examining the Role of Values on Mental Health Among Adolescents Across Regions in China. Front. Psychol..

[75] Ellermann C.R., Reed P.G. (2001). Self-Transcendence and Depression in Middle-Age Adults. West. J. Nurs. Res..

[76] Ormel J., de Jonge P. (2011). Unipolar Depression and the Progression of Coronary Artery Disease: Toward an Integrative Model. Psychother. Psychosom..

[77] Dinga R., Marquand A.F., Veltman D.J., Beekman A.T.F., Schoevers R.A., van Hemert A.M., Penninx B.W.J.H., Schmaal L. (2018). Predicting the Naturalistic Course of Depression from a Wide Range of Clinical, Psychological, and Biological Data: A Machine Learning Approach. Transl. Psychiatry.

[78] Horackova K., Kopecek M., Machů V., Kagstrom A., Aarsland D., Motlova L.B., Cermakova P. (2019). Prevalence of Late-Life Depression and Gap in Mental Health Service Use across European Regions. Eur. Psychiatr..

[79] Blazer D.G. (2003). Depression in Late Life: Review and Commentary. J. Gerontol. Ser. A Biol. Sci. Med. Sci..

[80] Hintsanen M., Pulkki-Råback L., Juonala M., Viikari J.S.A., Raitakari O.T., Keltikangas-Järvinen L. (2009). Cloninger’s Temperament Traits and Preclinical Atherosclerosis: The Cardiovascular Risk in Young Finns Study. J. Psychosom. Res..

[81] Bezgin C.H., Bezgin T., Kesebir S. (2016). Temperament and Character Profiles and Psychiatric Comorbidities in Patients With Coronary Artery or Valvular Heart Disease: Relationship With Cardiac Disease Severity. J. Clin. Med. Res..

[82] Lahat A., Benson B.E., Pine D.S., Fox N.A., Ernst M. (2016). Neural Responses to Reward in Childhood: Relations to Early Behavioral Inhibition and Social Anxiety. Soc. Cogn. Affect. Neurosci..

[83] Sher L. (2005). Type D Personality: The Heart, Stress, and Cortisol. QJM: Int. J. Med..

[84] Garcia D., Anckarsäter H., Lundström S. (2013). Self-Directedness and Cooperativeness, Psychosocial Dysfunction and Suffering in ESSENCE. Sci. World J..

[85] Steingrimsson S., Carlsen H.K., Lundström E., Lundström S., Nilsson T. (2020). Problematic Alcohol and Drug Use Is Associated with Low Self-Directedness and Cooperativeness. Eur. Addict. Res..

[86] de Miranda Azevedo R., Roest A.M., Hoen P.W., de Jonge P. (2014). Cognitive/Affective and Somatic/Affective Symptoms of Depression in Patients with Heart Disease and Their Association with Cardiovascular Prognosis: A Meta-Analysis. Psychol. Med..

[87] Ossola P., Gerra M.L., De Panfilis C., Tonna M., Marchesi C. (2018). Anxiety, Depression, and Cardiac Outcomes after a First Diagnosis of Acute Coronary Syndrome. Health Psychol..

[88] Meyer T., Hussein S., Lange H.W., Herrmann-Lingen C. (2015). Anxiety Is Associated with a Reduction in Both Mortality and Major Adverse Cardiovascular Events Five Years after Coronary Stenting. Eur. J. Prev. Cardiol..

