# Supplementary material for: Psychopathology, Personality and Depression after Acute Coronary Syndrome: A Network Analysis in an Italian Population
Source: Diagnostics (Basel). 2023 Feb 28;13(5):915. doi: 10.3390/diagnostics13050915 (PMC10000947; doi:10.3390/diagnostics13050915)
Supplement: Supplementary file 1 [file diagnostics-13-00915-s001.zip › diagnostics-2132174-supplementary.pdf]

Supplementary materials

METHODS

S1.1 Questionnaires

The PRIME-MD [4] is a structured interview designed to diagnose mental disorders according to DSM-IV. It evaluates the presence of nine depressive symptoms over the last two weeks. Every symptom is rated on a 4-point scale from “not at all” to “most days” (e.g "*Thoughts that you would be better off dead or of hurting yourself in some way*”). In addition, it contains a specific section that evaluates daily functioning due to a possible depressive symptomatology from “not at all” to “extremely difficult”. The PRIME-MD demonstrated good specificity and sensitivity in detecting major depressive episode in primary care [5].

The HADS [6] is a self-administered tool of 14 items, rated on a five-point likert scale (0-4). It contains two subscale scores, the anxiety score (HADS-A) and the depressive score (HADS-D); The scale was specifically developed to evaluate depressive and anxiety symptoms in hospitalised subjects hence relies less on the patients’ somatic complains [7].

The PRIME-MD PHQ [8] is the first self-administered version of PRIME-MD, elaborated with the purpose of make it more suitable for the clinical practice [8]. The original version contains questions that assess the presence of somatoform disorder, mood disorders, anxiety disorders, eating disorders and alcohol disorders; another section was later added to screen problems about menstruation, pregnancy, childbirth, and recent psychosocial stressors. Differently from PRIME-MD, which can diagnose 18 different psychiatric disorders, this test groups some items into larger rubrics, assessing only 8 possible diagnoses. In the final section there is a question for whom checked off any problems of the test (“how difficult have these problems made it for you to do your work, take care of things at home, or get along with ither people?”) [4]. Each module can be used separately from others [9], hence in this project we not included those related to eating disorders, menstruation, pregnancy, childbirth, and the one on mood disorders because it was overlapping with the PRIME-MD, previously given.

All questions of the selected modules were included for the analysis, except for one about menstruation (item 1d of the section about somatoform disorder) and some about psychosocial stressors (items from 7d to 7j). Some modules, like that about anxiety, that focuses on two different diagnoses (panic disorder and other anxiety disorders), were grouped to a single item during analysis.

The TCI [10,11] is a self-report instrument of 240 items with forced-choice (true/false) designed to assess temperament and character. It loads on seven subscales; four measures temperament: novelty seeking (NS), harm avoidance (HA), reward dependence (RD), and persistence (P); three,

instead, evaluate the character: self-directedness (SD), cooperativeness (C), and self-transcendence (ST). Specifically, NS can be described like the tendency to respond with excitement to novel stimuli or potential reward or potential relief of punishment. HA is defined as a tendency to respond strongly to signals of aversive stimuli. RD is the capacity to activate intensely to signals of reward, in particular social rewards. These three dimensions are thought to be connected to specific neurotransmitter systems: NS with dopamine, HA with serotonine and RD with norepinephrine [10]. Finally, P can be intended as perseverance in own behaviors, despite frustration and fatigue. Regarding character dimensions, SD is the capacity to adapt own behaviours in accordance with chosen targets and values. C reflects personal tendency to tolerance, compassion, empathy and helpfulness. ST is the ability to accept ambiguity and uncertainty [12,13].

The TAS-20 [14,15] is a self-reported 20 items questionnaires organized into three different subscales: one about difficulty identifying feelings (DIF) (e.g. "*I have feelings that I can't quite identify*"), one about difficulty describing, communicating and expressing feelings to others (DDF) (e.g. "*It is difficult for me to find the right words for my feelings*"), one about externally oriented thinking (EOT) (e.g "*I prefer to analyze problems rather than just describe them*"). The three subscales can also be summed to obtain a total score. A TAS-20 total score major than 60 has been proposed to define someone as alexithymic.

Type D Personality was measured with the DS-14 [16], a self-reported questionnaire of 14 items divided into two subscales, developed to evaluate Negative Affectivity (NA) and Social Inhibition (SI). Each scale consists of seven items rated from zero to four. In particular, NA dimension evaluate tendency to experience feeling of sadness, dysphoria, anxiety and irritability (e.g. "*Feel unhappy*"), while SI refers to discomfort in social interactions, lack of social poise, tendency to avoid confrontation and non-expression of emotions (e.g. "*Keep other people at a distance*")

The DSQ-40 [17,18] is built on assumption that, although defense mechanisms act in unconscious way, these are associated with behaviours and beliefs which can be described [19]. This short version of the test contains two items for each of 20 defense mechanisms, and evaluates three styles of defence styles, namely mature, neurotic and immature, that can be seen in a hierarchically order in the base of their supposed maturity. Specifically, the mature style includes functional mechanisms and allows for a proper assessment of internal and external reality of the subject. Unlike the latter, the neurotic style's mechanisms alter the perception of internal reality without major alterations of the external, while the Immature style can lead to major alterations about both the two dimensions [20]. In conclusion, the first style is more often related to physical and mental health, while others two are more linked to psychopathology [18]. Examples of phrases contained in the test are "*If my boss bugged me, I might make a mistake in my work or work more slowly so as to get back at him*" or "*I always feel that someone I know is like a guardian angel*".

The Network approach to psychopathology [21] represents conditional dependence relationships between variables, displayed as “nodes” connected with “edges” that is lines of different thickness, depending on the strength of correlation. In network theory a dense network confers more vulnerability towards a disorder – for instance responders to antidepressant treatment had a more dense depression network than non-responders [22], while emotion networks with higher density may contribute to depression and anxiety [23].

Of note both depressive disorder and coronary heart disease are chronic and recurrent conditions so that multiple depressive episodes might have a progressive impact on heart health and similarly a long history of CHD might impact on the subjects ability to face and overcome stressors. In light of the circular pathogenesis between CHD and depression, the best way to study the phenomenon and overcoming the reciprocal influence is to explore their association using network analyses, that are particularly suited to explore complex dynamics.

More specifically, we investigated whether (a) the severity of state- and trait-like symptoms at baseline differed between those who developed depression and cardiovascular events/death; (b) the strength of the reciprocal associations between features were different, under a network of psychopathology theoretical framework.

RESULTS

S1.2 Symptom reduction

Table S1. State-like symptoms loadings for each network community

	Factor 1 Depression	Factor 2 Somatic	Factor 4 Anxiety	Factor 3 Agitation
PMDT0b<br>Prime MD depressed mood	0.281			
HADST07<br>HADS relax	0.264			
PMDT0a<br>Prime MD apathy anhedonia	0.22			
PMDT0g<br>Prime MD concentration difficulties	0.214			
HADST02	0.203			

HADS anhedonia				
HADST04<br>HADS fun	0.202			
PMDT0f<br>Prime MD feelings of being a failure	0.2			
PHQT04<br>PHQ nervousness	0.191			
HADST012<br>HADS optimism	0.184			
PMDT0h<br>Prime MD psychomotor retardation or agitation	0.175			
PMDT0d<br>Prime MD low energy	0.165			
PMDT0c<br>Prime MD sleep problems	0.144			
PMDT0e<br>Prime MD low or high appetite	0.132			
HADST014<br>HADS pleasure	0.127			
HADST09<br>HADS tension	0.126			
PMDT0i<br>Prime MD thoughts of death	0.088			
PHQT07a<br>PHQ health worries		0.128		
PHQT01l<br>PHQ gastrointestinal problems		0.243		
PHQT01j<br>PHQ tachicardia		0.223		
PHQT01h<br>PHQ dizziness		0.22		
PHQT01b<br>PHQ back pain		0.214		
PHQT01m<br>PHQ nausea		0.209		
PHQT01i<br>PHQ faint		0.197		
PHQT01k<br>PHQ out of breath		0.193		
PHQT01c<br>PHQ pain limbs and joints		0.156		
PHQT01a<br>PHQ stomach ache		0.148		
PHQT01e<br>PHQ pain during sex		0.146		
PHQT07c<br>PHQ no sex pleasure		0.143		
PHQT01f<br>PHQ headache		0.132		
PHQT01g<br>PHQ chestpain		0.114		
PHQT07b		0.072		

PHQ body image worries				
PHQT06<br>PHQ Alcohol		0.06		
PHQT02<br>PHQ anxiety			0.49	
PHQT03<br>PHQ anxiety symptoms			0.49	
HADST013<br>HADS panic				0.398
HADST05<br>HADS worries				0.392
HADST06<br>HADS good mood				0.387
HADST08<br>HADS psychomotor retardation				0.319
HADST011<br>HADS restlessness				0.301
HADST03<br>HADS fear				0.267
HADST01<br>HADS nervousness				0.256
HADST010<br>HADS lack of self care				0.247

+++++++ NETWORKS WITH ALL PATIENTS ++++++

++ SYMPTOMS ALL

S2. Networks of the baseline state- and trait-measures in the whole sample

The network estimation of the state-like symptoms clusters with a Bayesian approach in the whole sample identified two separate communities, namely “Agitation” and “Depression, anxiety and somatic symptoms” (**Figure 1**).

Figure S1. Network of state-like measures with Bayesian estimation and walktrap community detection in the whole sample at baseline

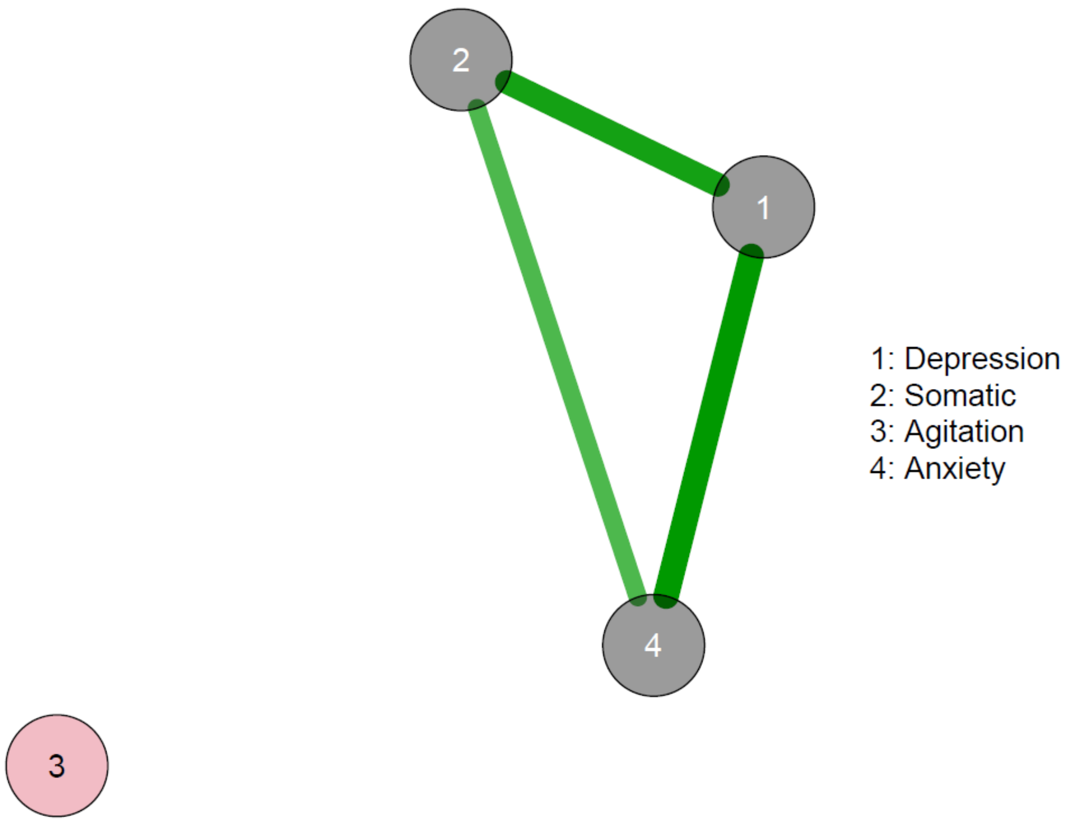

Note. Each node corresponds to a symptom cluster identified in the previous symptom reduction. Nodes are connected with lines (edges) that represent the strength of association between nodes. Green edges suggest a positive association whereas red edges suggest a negative association. The thickness of the edges represents the edge weight which is an indication of the strength of the association. The thicker the edge, the greater the weight, the stronger the association. In this network, all edges connecting the nodes are green (positive correlation), with their width indicating the strength of the connection. Node colors indicate the community of the nodes: pink for Agitation and grey for Depression, anxiety and somatic symptoms.

Anxiety, Depression and Somatic symptoms were positively correlated, with the strongest connection between Depression and Anxiety, while no connections were observed between the two communities. The network WAM is reported in **Table S2**. Centrality measures are reported in **Table S3**. The Depression node displayed the highest closeness centrality, strength and expected influence, followed by the Anxiety and Somatic nodes.

Table S2. Weighted Adjacency Matrix of the state-like symptoms network with Bayesian estimation in the whole sample

	Depression	Somatic	Agitation	Anxiety

Depression		0.3		0.33
Somatic	0.3			0.23
Agitation				
Anxiety	0.33	0.23		

Table S3. Centrality measures of the state-like symptoms network with Bayesian estimation in the whole sample

	Betweenness	Closeness	Strength	Expected influence
Depression	0	0.1575803	0.6313585	0.6313585
Somatic	0	0.1305058	0.5321947	0.5321947
Agitation	0	NA	0.00	0.00
Anxiety	0	0.1350384	0.5577832	0.5577832

++ PERSONALITY ALL

The network estimation of baseline trait-like features in the whole sample identified four communities, corresponding to the four scales used for the assessment (**Figure S2**).

Figure S2. Network of trait-like measures with Bayesian estimation and walktrap community detection in the whole sample at baseline

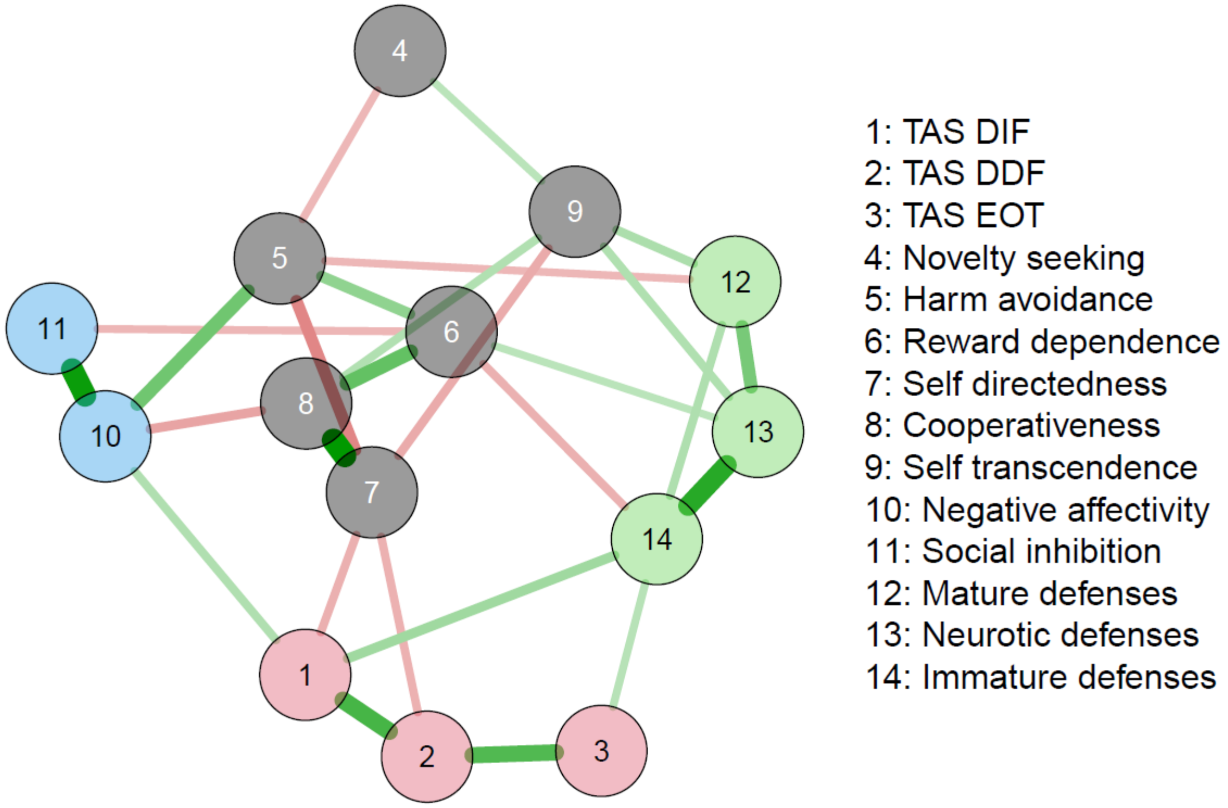

Note. Each node corresponds to a subscale of the trait-like measures. Nodes are connected with lines (edges) that represent the strength of association between nodes. Green edges suggest a

positive association whereas red edges suggest a negative association. The thickness of the edges represents the edge weight which is an indication of the strength of the association. The thicker the edge, the greater the weight, the stronger the association. Node colors indicate the community to which the nodes belong: pink for Defenses, green for the TAS, grey for Personality traits and light blue for type D personality traits.

The TAS community was positively associated with the DSQ-40 and the DS-14, and negatively associated with the TCI. In the DSQ-40 community, Mature defenses positively correlated with Self-transcendence and negatively correlated with Harm avoidance; Neurotic defenses positively correlated with Reward dependence and Self-transcendence, and Immature defenses positively correlated with the TAS DIF and the TAS EOT and negatively correlated with Reward dependence. In the TCI community, Harm avoidance positively correlated with the DS-14 Negative affectivity; Reward dependence positively correlated with Neurotic defenses and negatively correlated with Immature defenses and DS-14 Social Inhibition; Self-directedness negatively correlated with the TAS DIF and DDF; Cooperativeness negatively correlated with DS-14 Negative affectivity; Self-transcendence positively correlated with Mature and Neurotic defenses. In the DS-14 community, Negative affectivity negatively correlated with the TCI Reward dependence and Social Inhibition negatively correlated with the TCI Cooperativeness. The WAM of the network is displayed in **Table S4**. The strongest connections were that between the TCI Self-directedness and Cooperativeness, followed by that between the DS-14 subscales, and the connection between the DSQ-40 Immature and Neurotic defenses. The TCI Self-directedness displayed the greatest closeness centrality and strength (**Table S5**).

Table S4. Weighted Adjacency Matrix of the trait-like features network with Bayesian estimation in the whole sample

	TAS DIF	TAS DDF	TAS EOT	TCI NS	TCI HA	TCI RD	TCI SD	TCI C	TCI ST	PERS D NA	PERS D SI	DSQ Mature	DSQ Neurotic	DSQ Immature
TAS DIF		0.35					-0.15			0.15				0.18
TAS DDF	0.35		0.33				-0.15							
TAS EOT		0.33												0.14
TCI NS					-0.14				0.13					
TCI HA				-0.14		0.21	-0.23			0.27		-0.14		
TCI RD					0.21			0.3			-0.14		0.13	-0.15

TCI SD	-0.15	-0.15			-0.23			0.48	-0.16					
TCI C						0.3	0.48		0.15	-0.17				
TCI ST				0.13			-0.16	0.15				0.16	0.15	
PERS D NA	0.15				0.27			-0.17			0.46			
PERS D SI						-0.14				0.46				
DSQ Mature				-0.14					0.16				0.26	0.15
DSQ Neurotic						0.13			0.15			0.26		0.41
DSQ Immature	0.18		0.14			-0.15						0.15	0.41	

Table S5. Centrality measures of the state-like symptoms network with Bayesian estimation in the whole sample

	Betweenness	Closeness	Strength	Expected influence
TAS DIF	9	0.008426270	0.8283915	0.530857799
TAS DDF	8	0.007579005	0.8271366	0.533473370
TAS EOT	0	0.006463711	0.4661718	0.466171812
TCI NS	0	0.005549520	0.2655513	-0.006209932
TCI HA	12	0.009177746	0.9825125	-0.021111790
TCI RD	6	0.008605966	0.9206569	0.349805780
TCI SD	15	0.009259239	1.1678015	-0.202486212
TCI C	9	0.009082378	1.0989456	0.752622586
TCI ST	5	0.007512733	0.7432135	0.423699598
PERS D NA	11	0.008445095	1.0558108	0.709487757
PERS D SI	0	0.007213137	0.6009660	0.325362744
DSQ Mature	1	0.007185501	0.6991274	0.426841126
DSQ Neurotic	7	0.007760055	0.9426334	0.942633372
DSQ Immature	8	0.008119366	1.0235847	0.728336892

++++++++ WITH AND WITHOUT DEPRESSION (MDE) ++++++++++++++

++ SYMPTOMS

Table S6. Weighted Adjacency Matrix of the state-like symptoms network with Bayesian estimation in those with and without MDE at follow-up

	With MDE					Without MDE			
	Depression	Somatic	Agitation	Anxiety		Depression	Somatic	Agitation	Anxiety
Depression				0.4			0.33		
Somatic			0.25			0.33			0.29
Agitation		0.25							
Anxiety	0.4						0.29		

Table S7. Centrality measures of the state-like symptoms network with Bayesian estimation in those without MDE at follow-up

	With MDE					Without MDE			
	Betweenness	Closeness	Strength	Expected influence		Betweenness	Closeness	Strength	Expected influence
Depression	0	0.3955526	0.3955526	0.3955526		0	0.1047788	0.3270869	0.3270869
Somatic	0	NA	0.2527618	0.2527618		1	0.1541634	0.6186889	0.6186889
Agitation	0	NA	0.2527618	0.2527618		0	NA	0.00	0.00
Anxiety	0	0.3955526	0.3955526	0.3955526		0	0.1008476	0.2916021	0.2916021

++ PERSONALITY

YES DEPRE

Table S8. Weighted Adjacency Matrix of the trait-like features network with Bayesian estimation in those with MDE at follow-up

	TAS DIF	TAS DDF	TAS EOT	TCI NS	TCI HA	TCI RD	TCI SD	TCI C	TCI ST	PERS D NA	PERS D SI	DSQ Mature	DSQ Neurotic	DSQ Immature
TAS DIF		0.51								0.31				
TAS DDF	0.51									-0.28				
TAS EOT									-0.31					
TCI NS														
TCI HA							-0.32			0.35				
TCI RD												0.27		
TCI SD					-0.32			0.46	-0.31					
TCI C							0.46		0.34					-0.32
TCI ST			-0.31				-0.31	0.34						
PERS D NA	0.31	-0.28			0.35						0.51			
PERS D SI										0.51				

DSQ Mature						0.27								
DSQ Neurotic														0.52
DSQ Immature								-0.32					0.52	

Table S9. Centrality measures of the trait-like features network with Bayesian estimation in those with MDE at follow-up

	Betweenness	Closeness	Strength	Expected influence
TAS DIF	0	0.010354643	0.8216566	0.82165657
TAS DDF	0	0.010039114	0.7927230	0.23514468
TAS EOT	0	0.009569657	0.3147744	-0.31477439
TCI NS	0	NA	0.00	0.00
TCI HA	24	0.015716229	0.6657844	0.03268032
TCI RD	0	NA	0.2685819	0.26858193
TCI SD	25	0.016537275	1.0871176	-0.15745436
TCI C	16	0.015093743	1.1209481	0.49004760
TCI ST	9	0.013174354	0.9611746	-0.27984210
PERS D NA	23	0.013846817	1.4460343	0.88845592
PERS D SI	0	0.011128943	0.5102901	0.51029006
DSQ Mature	0	NA	0.2685819	0.26858193
DSQ Neurotic	0	0.009465376	0.5231013	0.52310127
DSQ Immature	9	0.011306702	0.8385515	0.20765100

NO DEPRE PERSONALITY

Table S10. Weighted Adjacency Matrix of the trait-like features network with Bayesian estimation in those without MDE at follow-up

	TAS DIF	TAS DDF	TAS EOT	TCI NS	TCI HA	TCI RD	TCI SD	TCI C	TCI ST	PERS D NA	PERS D SI	DSQ Mature	DSQ Neurotic	DSQ Immature
TAS DIF		0.29					-0.17							0.19
TAS DDF	0.29		0.33				-0.18							
TAS EOT		0.33												
TCI NS					-0.16				0.18					
TCI HA				-0.16		0.2	-0.19			0.25		-0.15		
TCI RD					0.2			0.34					0.14	-0.17
TCI SD	-0.17	-0.18			-0.19			0.49						
TCI C						0.34	0.49			-0.15				

TCI ST				0.18										
PERS D NA					0.25			-0.15			0.44	0.17		
PERS D SI										0.44				
DSQ Mature					-0.15					0.17			0.28	
DSQ Neurotic						0.14						0.28		0.39
DSQ Immature	0.19					-0.17							0.39	

Table S11. Centrality measures of the trait-like features network with Bayesian estimation in those without MDE at follow-up

	Betweenness	Closeness	Strength	Expected influence
TAS DIF	6	0.006965843	0.6461634	0.31564457
TAS DDF	12	0.006831660	0.8026402	0.44248939
TAS EOT	0	0.005467455	0.3285588	0.32855881
TCI NS	12	0.005814278	0.3376326	0.01769333
TCI HA	25	0.009134209	0.9506499	-0.06072256
TCI RD	7	0.008312373	0.8492900	0.51668654
TCI SD	24	0.008663894	1.0252353	-0.04975827
TCI C	14	0.008441328	0.9778427	0.68075212
TCI ST	0	0.004174772	0.1776630	0.17766299
PERS D NA	13	0.007618295	1.0045244	0.70743387
PERS D SI	0	0.006309859	0.4408653	0.44086532
DSQ Mature	7	0.007109326	0.6021365	0.29502726
DSQ Neurotic	7	0.007246240	0.8183220	0.81832200
DSQ Immature	6	0.007230505	0.7459610	0.41335757

Table S12. Edge differences in the trait-like features network with Bayesian estimation between those with and without depression at follow-up

	TAS DIF	TAS DDF	TAS EOT	TCI NS	TCI HA	TCI RD	TCI SD	TCI C	TCI ST	PERS D NA	PERS D SI	DSQ Mature	DSQ Neurotic	DSQ Immature
TAS DIF		-0.289								-0.303				
TAS DDF	-0.289									0.439				
TAS EOT														
TCI NS														
TCI HA									0.380					
TCI RD														
TCI SD														

TCI C														
TCI ST					0.380				-2.22e-16					
PERS D NA	-0.303	0.439										0.367		
PERS D SI														
DSQ Mature										0.367				
DSQ Neurotic														
DSQ Immature														

++++++++ WITH AND WITHOUT CARDIAC EVENTS AND DEATHS (MACE) ++++++++++++++

++ SYMPTOMS

Table S13. Weighted Adjacency Matrix of the state-like symptoms network with Bayesian estimation in those without MACE at follow-up

	**With MACE**					**Without MACE**			
	Depression	Somatic	Agitation	Anxiety		Depression	Somatic	Agitation	Anxiety
Depression				0.48			0.32		0.28
Somatic						0.32			0.26
Agitation									
Anxiety	0.48					0.28	0.26		

Table S14. Centrality measures of the state-like symptoms network with Bayesian estimation in those without MACE at follow-up

	**With MACE**					**Without MACE**			
	Betweenness	Closeness	Strength	Expected influence		Betweenness	Closeness	Strength	Expected influence
Depression	0	0.4771994	0.4771994	0.4771994		0	0.1490115	0.5981301	0.5981301
Somatic	0	NA	0.00	0.00		0	0.1416041	0.5728287	0.5728287

Agitation	0	NA	0.00	0.00		0	NA	0.00	0.00
Anxiety	0	0.4771994	0.4771994	0.4771994		0	0.1340831	0.5375233	0.5375233

++ PERSONALITY

YES MACE

Table S15. Weighted Adjacency Matrix of the trait-like features network with Bayesian estimation in those without MDE at follow-up

	TAS DIF	TAS DDF	TAS EOT	TCI NS	TCI HA	TCI RD	TCI SD	TCI C	TCI ST	PERS D NA	PERS D SI	DSQ Mature	DSQ Neurotic	DSQ Immature
TAS DIF														
TAS DDF			0.37											
TAS EOT		0.37							-0.28					
TCI NS									0.27			0.3		
TCI HA											0.31			
TCI RD											0.29			-0.4
TCI SD								0.52						
TCI C							0.52		0.3					
TCI ST			-0.28	0.27				0.3						
PERS D NA											0.3			
PERS D SI					0.31	0.29				0.3				0.29
DSQ Mature				0.3										
DSQ Neurotic														0.42
DSQ Immature						-0.4					0.29		0.42	

Table S16. Centrality measures of the trait-like features network with Bayesian estimation in those without MDE at follow-up

	Betweenness	Closeness	Strength	Expected influence
TAS DIF	0	NA	0.00	0.00
TAS DDF	0	0.01866861	0.3675087	0.3675087
TAS EOT	5	0.02502457	0.6457142	0.0893032
TCI NS	5	0.02491899	0.5776032	0.5776032
TCI HA	0	NA	0.3075112	0.3075112
TCI RD	0	NA	0.6888415	-0.1066940
TCI SD	0	0.02044764	0.5209258	0.5209258
TCI C	5	0.02544070	0.8170827	0.8170827
TCI ST	12	0.03427318	0.8482675	0.2918566

PERS D NA	0	NA	0.2950537	0.2950537
PERS D SI	7	NA	1.1885054	1.1885054
DSQ Mature	0	0.01766980	0.3036980	0.3036980
DSQ Neurotic	0	NA	0.4190031	0.4190031
DSQ Immature	4	NA	1.1116376	0.3161021

NO MACE cardiac PERS

Table S17. Weighted Adjacency Matrix of the trait-like features network with Bayesian estimation in those without MDE at follow-up

	TAS DIF	TAS DDF	TAS EOT	TCI NS	TCI HA	TCI RD	TCI SD	TCI C	TCI ST	PERS D NA	PERS D SI	DSQ Mature	DSQ Neurotic	DSQ Immature
TAS DIF		0.36				0.17								0.21
TAS DDF	0.36		0.34											
TAS EOT		0.34											-0.14	0.15
TCI NS					-0.17									
TCI HA				-0.17		0.2	-0.24			0.27				
TCI RD	0.17				0.2			0.31			-0.26			
TCI SD					-0.24			0.45	-0.18					
TCI C						0.31	0.45			-0.18	0.14			
TCI ST							-0.18					0.2	0.16	
PERS D NA					0.27			-0.18			0.48			
PERS D SI						-0.26		0.14		0.48				
DSQ Mature									0.2				0.27	
DSQ Neurotic			-0.14						0.16			0.27		0.42
DSQ Immature	0.21		0.15										0.42	

Table S18. Centrality measures of the trait-like features network with Bayesian estimation in those without MDE at follow-up

	Betweenness	Closeness	Strength	Expected influence
TAS DIF	24	0.008248111	0.7303616	0.73036156
TAS DDF	8	0.006703242	0.6984821	0.69848209
TAS EOT	3	0.006136645	0.6298716	0.34744753
TCI NS	0	0.004960658	0.1652745	-0.16527448
TCI HA	12	0.007753160	0.8735701	0.07249082
TCI RD	29	0.008930429	0.9406763	0.41291006
TCI SD	17	0.008033569	0.8628924	0.03456783
TCI C	12	0.008188638	1.0784908	0.71303011
TCI ST	12	0.007067193	0.5400797	0.18228538
PERS D NA	2	0.006842487	0.9289131	0.56345240
PERS D SI	6	0.006940771	0.8811086	0.35334233
DSQ Mature	0	0.006142114	0.4767874	0.47678740
DSQ Neurotic	9	0.006654577	0.9984038	0.71597979
DSQ Immature	6	0.006920713	0.7776119	0.77761193

Table S19. Edge differences in the trait-like features network with Bayesian estimation between those with and without MACE at follow-up

	TAS DIF	TAS DDF	TAS EOT	TCI NS	TCI HA	TCI RD	TCI SD	TCI C	TCI ST	PERS D NA	PERS D SI	DSQ Mature	DSQ Neurotic	DSQ Immature
TAS DIF														
TAS DDF														
TAS EOT														
TCI NS														
TCI HA														
TCI RD											-0.554			
TCI SD								-0.206						
TCI C							-0.206							
TCI ST														
PERS D NA														
PERS D SI						-0.554								
DSQ Mature														
DSQ Neurotic														
DSQ Immature														

1. Kim, J.M.; Stewart, R.; Kang, H.J.; Kim, S.Y.; Kim, J.W.; Lee, H.J.; Lee, J.Y.; Kim, S.W.; Shin, I.S.; Kim, M.C.; et al. Long-Term Cardiac Outcomes of Depression Screening, Diagnosis and

Treatment in Patients with Acute Coronary Syndrome: The DEPACS Study. *Psychological medicine* **2021**, *51*, 964–974, doi:10.1017/S003329171900388X.
2. Yuan, M.Z.; Fang, Q.; Liu, G.W.; Zhou, M.; Wu, J.M.; Pu, C.Y. Risk Factors for Post-Acute Coronary Syndrome Depression: A Meta-Analysis of Observational Studies. *The Journal of cardiovascular nursing* **2019**, *34*, 60–70, doi:10.1097/JCN.0000000000000520.
3. Disner, S.G.; Beevers, C.G.; Haigh, E.A.P.; Beck, A.T. Neural Mechanisms of the Cognitive Model of Depression. *Nature reviews. Neuroscience* **2011**, *12*, 467–477, doi:10.1038/NRN3027.
4. Spitzer, R.L.; Williams, J.B.W.; Johnson, J.G.; Kroenke, K.; Linzer, M.; Degruy, F.V.; Brody, D.; Hahn, S.R. Utility of a New Procedure for Diagnosing Mental Disorders in Primary Care: The PRIME-MD 1000 Study. *JAMA: The Journal of the American Medical Association* **1994**, *272*, 1749–1756, doi:10.1001/jama.1994.03520220043029.
5. Spitzer, R.L.; Kroenke, K.; Williams, J.B.W. Validation and Utility of a Self-Report Version of PRIME-MD. *Primary Care Companion to the Journal of Clinical Psychiatry* **2000**, *2*, 31.
6. Zigmond, A.S.; Snaith, R.P. The Hospital Anxiety and Depression Scale. *Acta Psychiatrica Scandinavica* **1983**, doi:10.1111/j.1600-0447.1983.tb09716.x.
7. Doyle, F.; McGee, H.M.; De La Harpe, D.; Shelley, E.; Conroy, R. The Hospital Anxiety and Depression Scale Depression Subscale, but Not the Beck Depression Inventory-Fast Scale, Identifies Patients with Acute Coronary Syndrome at Elevated Risk of 1-Year Mortality. *Journal of Psychosomatic Research* **2006**, *60*, 461–467, doi:10.1016/j.jpsychores.2005.09.004.
8. Spitzer, R.L.; Kroenke, K.; Williams, J.B.W. Validation and Utility of a Self-Report Version of PRIME-MD. *Primary Care Companion to the Journal of Clinical Psychiatry* **2000**, *2*, 31.
9. Kroenke, K.; D, M.; Spitzer, R.L.; D, M.; Williams, J.B.W.; W, D.S.; Löwe, B.; Ph, D. The Patient Health Questionnaire Somatic , Anxiety , and Depressive Symptom Scales : A Systematic Review. *General Hospital Psychiatry* **2010**, *32*, 345–359, doi:10.1016/j.genhosppsych.2010.03.006.
10. Cloninger, C.R.; Svrakic, D.M.; Przybeck, T.R. A Psychobiological Model of Temperament and Character. *Archives of General Psychiatry* **1993**, *50*, 975–990, doi:10.1001/archpsyc.1993.01820240059008.
11. Cloninger, C.R.; Przybeck, T.R.; Svrakic, D.M.; Wetzel, R.D. The Temperament and Character Inventory (TCI): A Guide to Its Development and Use Center for Psychobiology of Personality , Washington. **1994**.
12. Mikolajczyk, Z.; Zietek, J.; Samochowiec, A.; Samochowiec, J. Personality Dimensions Measured Using the Temperament and Character Inventory (TCI) and NEO-FFI on a Polish Sample. *International journal of methods in psychiatric research* **2008**, *17 Suppl 4*, 210–291, doi:10.1002/mpr.264.
13. Ossola, P.; Generali, I.; Schito, G.; De Panfilis, C.; Tonna, M.; Gerra, M.L.; Marchesi, C. Temperament and Depression After a First Acute Coronary Syndrome. *The Journal of nervous and mental disease* **2019**, *207*, 277–283, doi:10.1097/NMD.0000000000000964.
14. Bagby, R.M.; Taylor, G.J.; Parker, J.D.A. The Twenty-Item Toronto Alexithymia Scale-II. Convergent, Discriminant, and Concurrent Validity. *Journal of Psychosomatic Research* **1994**, doi:10.1016/0022-3999(94)90006-X.
15. Bagby, R.M.; Parker, J.D.A.; Taylor, G.J. The Twenty-Item Toronto Alexithymia Scale-I. Item Selection and Cross-Validation of the Factor Structure. *Journal of Psychosomatic Research* **1994**, doi:10.1016/0022-3999(94)90005-1.
16. Denollet, J. DS14: Standard Assessment of Negative Affectivity, Social Inhibition, and Type D Personality. *Psychosomatic Medicine* **2005**, doi:10.1097/01.psy.0000149256.81953.49.
17. Andrews, G.; Singh, M.; Bond, M. The Defense Style Questionnaire. *Journal of Nervous and Mental Disease* 1993, *181*, 246–256.
18. Bond, M. Empirical Studies of Defense Style: Relationships with Psychopathology and Change. *Harvard Review of Psychiatry* **2004**, *12*, 263–278, doi:10.1080/10673220490886167.
19. Wilkinson, W.W.; Ritchie, T.D. The Dimensionality of Defense-Mechanism Parcels in the Defense Style Questionnaire-40. *Psychological Assessment* **2015**, *27*, 326–331, doi:10.1037/pas0000051.

20. Ribadier, A.; Dorard, G.; Varescon, I. Personality and Defense Styles: Clinical Specificities and Predictive Factors of Alcohol Use Disorder in Women. *Journal of Psychoactive Drugs* **2016**, *48*, 384–392, doi:10.1080/02791072.2016.1234089.
21. Borsboom, D. A Network Theory of Mental Disorders. *World Psychiatry* **2017**, *16*, 5–13, doi:10.1002/wps.20375.
22. Van Borkulo, C.; Boschloo, L.; Borsboom, D.; Penninx, B.W.J.H.; Lourens, J.W.; Schoevers, R.A. Association of Symptom Network Structure with the Course of Longitudinal Depression. *JAMA Psychiatry* **2015**, *72*, 1219–1226, doi:10.1001/jamapsychiatry.2015.2079.
23. Shin, K.E.; Newman, M.G.; Jacobson, N.C. Emotion Network Density Is a Potential Clinical Marker for Anxiety and Depression: Comparison of Ecological Momentary Assessment and Daily Diary. *British Journal of Clinical Psychology* **2022**, *61*, 31–50, doi:10.1111/bjc.12295.
